# Integrated Clinical Trial and Molecular Profiling Reveals Immune Drivers of Chronic Hand Eczema

**DOI:** 10.1111/all.70263

**Published:** 2026-02-18

**Authors:** Perrine Gery, Rüçhan Ekren, Emilie Bérard, Aurélie Du‐Thanh, Hugo Rimet, Fatma Jendoubi, Vanina Lenief, Nadia Raison‐Peyron, Amel Bouznad, Maria P. Konstantinou, Jonathan Rioual, Lilian Basso, Juliette Mouiren, Manon Boussier, Brigitte Milpied, Cristina Bulai Livideanu, Louise Jaulent, Florence Hacard, Marine A. Lefevre, Audrey Nosbaum, Julien Seneschal, Marc Vocanson, Carle Paul, Nicolas Gaudenzio, Marie Tauber, the French Atopic Dermatitis Network from de GREAT Research group

**Affiliations:** ^1^ Centre International de Recherche en Infectiologie University of Lyon 1 and École Normale Supérieure de Lyon, Inserm U1111 Lyon France; ^2^ Infinity (Institut Toulousain des Maladies Infectieuses et Inflammatoires), Immception Lab, Inserm U1291 Toulouse University Toulouse France; ^3^ Department of Clinical Epidemiology and Public Health Toulouse University Hospital Toulouse France; ^4^ CERPOP, Inserm, University Toulouse III Paul Sabatier Toulouse France; ^5^ Department of Dermatology Saint‐Eloi Hospital, Montpellier University Montpellier France; ^6^ Department of Dermatology Larrey Hospital, Toulouse University Toulouse France; ^7^ CHU de Bordeaux, Dermatology and Pediatric Dermatology, National Reference Center for Rare Skin Disorders, Hôpital Saint‐André, UMR 5164 Bordeaux France; ^8^ University of Bordeaux, CNRS, ImmunoConcEpT, UMR 5164 Bordeaux France; ^9^ Department of Allergology and Clinical Immunology Hospices Civils de Lyon, Lyon Sud Hospital Pierre‐Bénite France

**Keywords:** chronic hand eczema, dupilumab, mixed immune signature, molecular profiling, randomized clinical trial

## Abstract

**Background:**

Chronic hand eczema (CHE) is a debilitating skin condition characterized by pain, itch, and chronic inflammation, with a complex multifactorial origin. Its diverse presentations, including overlapping traits of atopic dermatitis, contact dermatitis, and psoriasis, make diagnosis and treatment particularly challenging. The underlying immune mechanisms of CHE still need to be investigated.

**Methods:**

Here we conducted a comprehensive molecular profiling of CHE patients, enrolled without prior selection on etiology and morphology, and performed a phase 2b randomized, multicenter, double‐blind, placebo‐controlled clinical trial comparing dupilumab to placebo over 16 weeks.

**Results:**

We demonstrated that CHE patients may benefit from IL4Rα‐blockade, regardless of etiological factors or clinical presentation. Skin transcriptomic and serum profiles of CHE patients closely resemble those seen in both atopic dermatitis and psoriasis, with dysregulated genes affecting keratinocyte differentiation, leucocyte‐mediated immunity, cytokine signaling, and mixed type 1, 2, and 3 immunity. The clinical trial included 94 adults with moderate to severe CHE persisting for at least six months and resistant to potent topical corticosteroids. Compared to placebo control, 16‐week dupilumab treatment significantly improved clinical severity and quality of life of all CHE patients while largely restoring appropriate transcriptomic and proteomic programs related to skin barrier and immune homeostasis.

**Conclusion:**

These findings confirmed the involvement of not only type 2 but also type 1 and type 3 immunity across all CHE patients, and demonstrate that IL‐4Rα blockade may offer effective therapeutic perspectives for this highly burdensome condition, independent of an atopic dermatitis background.

## Graphical abstract

This study performed an unbiased molecular profiling of CHE patients across diverse etiologies to identify shared pathogenic drivers and evaluate the impact of IL‐4Rα blockade via dupilumab over 16 weeks. CHE shows a mixed immune signature involving type 1, 2, and 3 pathways with features of atopic dermatitis and psoriasis. Dupilumab significantly improves clinical severity and quality of life in moderate‐to‐severe CHE, regardless of AD background, and restores skin immune and barrier homeostasis by normalizing dysregulated transcriptomic and proteomic programs. AD, atopic dermatitis; CCL, C‐C motif chemokine ligand; CHE, chronic hand eczema; COL6A6, collagen type VI alpha 6 chain; CP, conventional psoriasis; IL, interleukin; LC–MS/MS, liquid chromatography–tandem mass spectrometry; MMP12, matrix metalloproteinase 12; mTLSS, modified total lesion symptom score; NOS2, nitric oxide synthase 2; SERPINB4, serpin family B member 4.

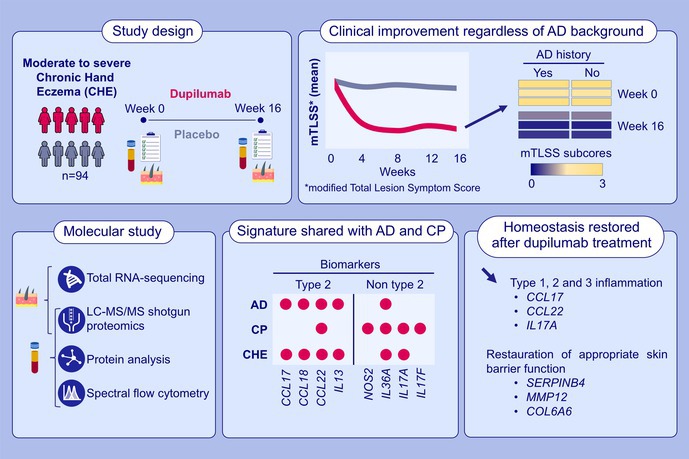

## 1 Introduction

Chronic hand eczema (CHE) is a common, highly painful, and itchy chronic inflammatory disease with a significant long‐lasting negative impact on quality of life. Due to its prevalence and impact, CHE has recently gained increasing attention in public health discussions. Currently, the only systemic treatment approved in Europe and Canada for patients refractory to topical therapies is the retinoid alitretinoin. Although its efficacy was demonstrated in clinical trials over 15 years ago [1], particularly in hyperkeratotic (thickened) forms of CHE, recent real‐world data report a median drug survival of approximately 100 days, likely due to a combination of limited effectiveness in some patients and adverse events [2]. While not life‐threatening, CHE causes significant suffering and can negatively affect work ability, career prospects, and social status [3]. Socioeconomically, CHE is associated with high direct treatment costs and indirect costs due to lost work hours [4, 5], imposing an economic burden on patients and society.

CHE is classically defined as an eczema/dermatitis that persists on the hands for more than three months or recurs twice or more within a 12‐month period [6]. In practice, CHE typically follows a persistent, rather than remitting and relapsing course. It is a highly heterogeneous condition thought to be of multifactorial origin, influenced by endogenous/inherited factors and exogenous/environmental exposures. A recognized risk factor for CHE development is a history of atopic dermatitis (AD) [7], a major type 2 immunity‐driven skin disease that is nowadays well‐managed worldwide using targeted treatments, including the anti‐IL4Rα dupilumab [8]. AD patients with mutations in the *FLG* gene, which is involved in the regulation of skin barrier and homeostasis, are particularly at risk for severe CHE. However, the presence of *FLG* mutations without associated AD does not significantly increase the risk of CHE [9, 10, 11]. Exogenous factors contributing to CHE include exposures that directly compromise the skin barrier, such as excessive water, irritants, detergents, and mechanical irritation from both occupational and domestic sources [12, 13, 14]. In addition to irritants, allergens (mainly chemicals) also play an important role [13, 14, 15].

CHE is empirically categorized based on both etiology (e.g., irritant contact dermatitis‐, allergic contact dermatitis‐, or AD‐related CHE) and clinical presentation (e.g., vesicular, fissured, and hyperkeratotic CHE) [16, 17]. However, there is no consistent correlation between etiology and clinical presentation. Despite allergy testing and thorough patient interviews on environmental triggers, over 20% of CHE patients remain unclassified, with most fitting into multiple categories [18, 19]. CHE may persist chronically even after an initial diagnosis of irritant contact dermatitis or allergic contact dermatitis and avoidance of triggering irritants and/or allergens. Additionally, distinguishing clinically hyperkeratotic CHE from palmar psoriasis can be very challenging in some patients, with skin histopathological examination often providing limited insight [20, 21]. These observational data suggest a complex interplay of immunological, environmental, and possibly genetic factors underlying the pathophysiology of CHE. The closely connected traits of AD, irritative or allergic contact dermatitis, and psoriasis in CHE patients make the condition particularly challenging to treat. Few studies [18, 22, 23, 24, 25] have aimed to molecularly profile CHE patients using proteomic or transcriptomic analyses and have often faced challenges in classifying patients. Some preliminary studies have explored the possibility of treating CHE with dupilumab, however in highly selected populations such as AD patients with hand eczema [26] and patients with the recurrent vesicular or chronic fissured subtypes of CHE [27]. Though, there is still a need to investigate the molecular nature of all CHE patients regardless of their atopic/allergic background or clinical presentation and to better understand the mechanism of action of dupilumab in this specific context.

We conducted a comprehensive profiling of CHE patients without preselection based on etiology and morphology to identify potential common molecular features and evaluated the consequences of blocking IL4Rα signaling during 16 weeks using dupilumab. This phase 2b randomized, multicenter, double‐blind, placebo‐controlled clinical trial showed that CHE patients share complex transcriptomic and proteomic signatures with those of AD and psoriasis patients, with dysregulated genes involved in keratinocyte differentiation, cytokine‐mediated signaling, type 1, 2, and 3 immunity. Compared to placebo control, 16‐week dupilumab treatment significantly improved clinical severity and quality of life of CHE patients, with a global restoration of appropriate transcriptomic and proteomic programs related to skin barrier and immune homeostasis.

## 2 Results

### 2.1 Subhead 1: Chronic Hand Eczema Is a Highly Heterogeneous Disease: Baseline Patient's Clinical Presentations

**FIGURE 1:**
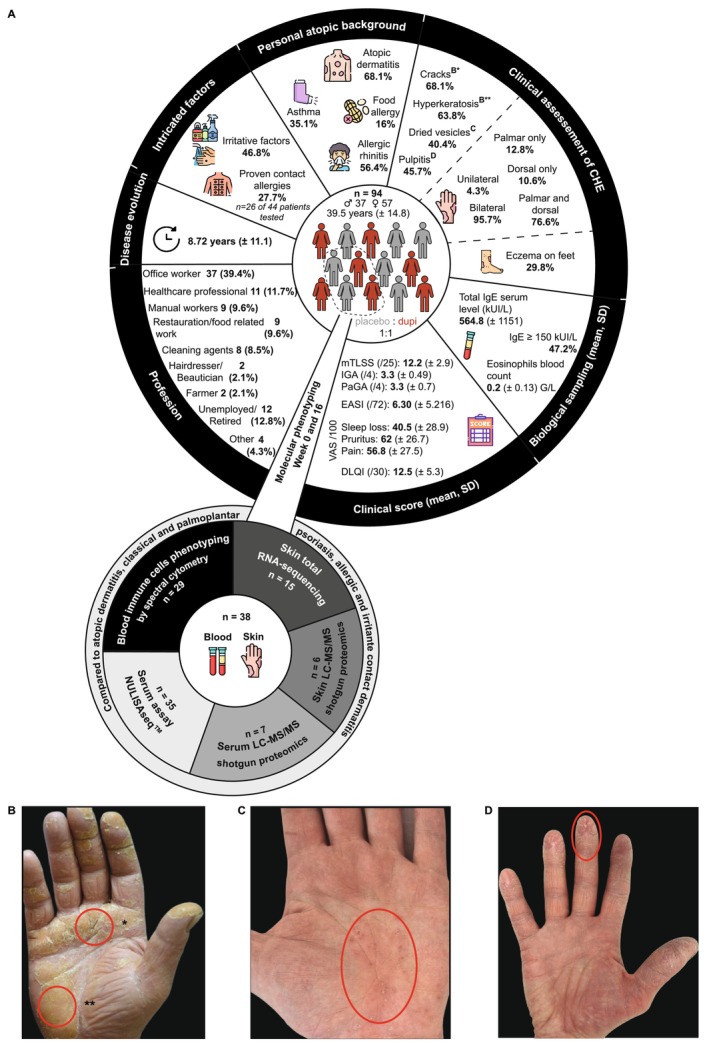
Baseline characteristics of patients included and CHE disease history. (A) Description of the chronic hand eczema (CHE) patients cohort at baseline. The largest round shows demographic characteristics (gender, age, profession), personal history of atopy, history of CHE disease (time since diagnosis, intricate causative factors), clinical severity and biological assessments. Regarding foot involvement at baseline, the specification of whether the lesions were plantar is not available. One third of the patients (*n* = 38) were included in the translational study, involving molecular phenotyping at baseline and after dupilumab treatment at week 16. Patients in gray represent the placebo group, while those in red represent the dupilumab group. The small circle describes the techniques used on patients' blood and skin samples: Skin bulk RNA‐sequencing, blood spectral cytometry, skin and serum proteomic using LC–MS/MS (shotgun proteomic) and serum assay using NULISAseq. CHE patients were compared to healthy controls and patients with atopic dermatitis (AD), classical psoriasis vulgaris (CP), palmoplantar psoriasis (PPPso), allergic contact dermatitis (ACD), and irritant contact dermatitis (ICD). (B–D) Representative hand pictures of the different types of lesions found in CHE patients. Cracks (B*), hyperkeratosis (B**), dried vesicles (C) and pulpitis (D) are represented. LC–MS/MS, Liquid chromatography coupled to tandem mass spectrometry.

Ninety‐four patients (57 females, 60.6%) with a mean age of 39.5 (standard deviation, SD: 14.8 years) were included in the randomized clinical trial (see Figure S1 for the flow chart of the study). Office work was the most represented profession (*n* = 37, 39.4%) followed by healthcare professionals (*n* = 11, 11.7%), manual workers such as construction workers, mechanics and plumbers (*n* = 9, 9.6%) and restauration/food related work (*n* = 9, 9.6%) (Figure 1A). A history of AD (defined as a diagnosis of AD including mild or intermittent forms, made prior to the diagnosis of CHE), asthma, allergic rhinitis, allergic conjunctivitis and food allergy was present in 68.1% (*n* = 64), 35.1% (*n* = 33), 56.4% (*n* = 53), 27.7% (*n* = 26) and 16.0% (*n* = 15) of patients, respectively (Figure 1A). An allergic contact dermatitis, confirmed by relevant positive patch testing, was identified in 27.7% of patients (*n* = 26 out of 44 patients tested from the overall cohort of 94). Irritation factors, gathered through patient interviews, were reported in 46.8% of cases (*n* = 44 out of 94) and were associated with work or leisure activities (Figure 1A). Notably, since patch testing was not mandatory for inclusion in the clinical trial, the proportion of relevant positive patch tests might be underestimated. CHE persisted for 8.72 years (SD: 11.1), with a chronicity of lesions. All patients received potent or highly potent topical corticosteroids and 34.4% (*n* = 31) topical calcineurin inhibitors. Only 18.1% (*n* = 17) of patients had previously participated in a therapeutic patient education program for CHE. The first systemic treatment received by patients prior to entering the clinical trial was alitretinoin (27.7%, *n* = 26, Table S1). CHE involved both hands in 95.7% of patients (*n* = 90) with the palm and the back of the hand concomitantly affected in 76.6% (*n* = 72) (Figure 1A). Cracks were the most frequent clinical presentation (68.1%, *n* = 64), followed by hyperkeratosis (63.8%, *n* = 60), pulpitis (45.7%, *n* = 43) and vesicles (40.4%, *n* = 38) (Figure 1B–D). Only 17 patients had only one clinical sign among the five (18.1%). Nails were affected in 35.1% of patients (*n* = 33). Feet were also affected in 29.8% of patients (*n* = 28) (Figure 1A, Table S2).

Regarding clinical severity at baseline, the mean mTLSS (modified Total Lesion Symptom Score/25), IGA (Investigator Global Assessment/4), PaGA (Patient Global Assessment/4) and VAS (Visual Analogical Scales/100) for pruritus, pain and sleep loss were respectively 12.2 (SD: 2.93), 3.3 (SD: 0.49), 3.3 (SD: 0.66), 62.0 (SD: 26.70), 56.8 (SD: 27.53) and 40.5 (SD: 28.85) (Figure 1A). The mean EASI (Eczema Area and Severity Index/72, *n* = 62), reflecting eczema on the hands, wrists or other limited body surface area, was 6.30 (SD: 5.22) (Figure 1A). The quality of life of CHE patients was considerably impaired with a mean DLQI (Dermatology Life Quality Index/30) of 12.5 (SD: 5.28) as well as the activity and the work productivity assessed with the WPAI (Work Productivity and Activity Impairment) questionnaire. Patients reported an average work time loss of 9.4% (SD: 27.10), a health‐related impairment while working of 38.1% (SD: 25.41), an overall work impairment due to health of 44.1% (SD: 28.45) and a health‐related activity impairment of 54.3% (SD: 25.70) (Figure 1A, Table S2).

Regarding biological baseline measures, blood eosinophils count at baseline was in normal ranges in all patients (*n* = 93) with a mean of 0.20 G/L (SD: 0.13), total IgE serum level was above normal range (> 150 kUI/L, reflecting an active atopic background) in 47.2% of patients (*n* = 42) with a mean of 564.83 (SD: 1151.15) in the whole cohort (*n* = 89) (Figure 1A, Table S2).

### 2.2 Subhead 2: Chronic Hand Eczema Can Be Efficiently Reversed With Dupilumab Regardless of Atopic Dermatitis Background

**FIGURE 2:**
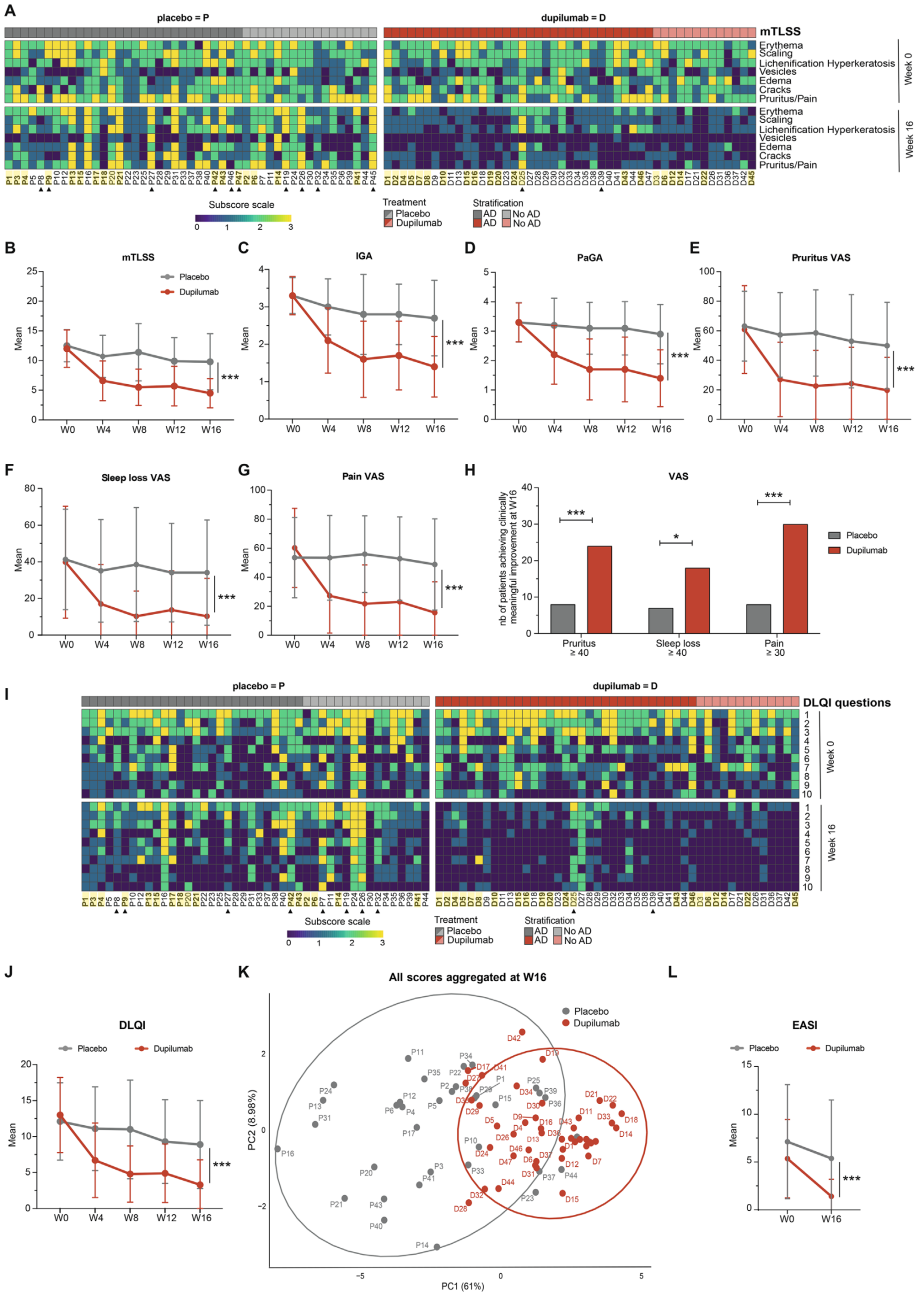
Chronic hand eczema can be efficiently reversed with dupilumab regardless of atopic dermatitis background. (A) Heat map representation of modified Total Lesion Symptom Score (mTLSS) evolution under dupilumab treatment or placebo. Erythema, scaling, lichenification/hyperkeratosis, vesicles, edema, cracks and pruritus/pain, were evaluated for each patient at week 0 and 16 after the beginning of the treatment. Rows and columns represent each mTLSS subscore and patients, respectively. Patient numbers are reported at the bottom of the figure with patients included in the translational study highlighted in yellow. The subscore scale ranges from blue (absent) to yellow (severe). (B–G) Clinical scores evolution under dupilumab treatment or placebo. Total mTLSS (B), Investigator Global Assessment (IGA) (C), Patient Global Assessment (PaGA) (D) and visual analogical scales (VAS) evaluating pruritus (E), sleep loss (F) and pain (G) were assessed at weeks 0, 4, 8, 12 and 16. Data are presented as mean ± standard deviation. (H) Number of patients achieving clinically meaningful improvement in symptoms at week 16. Week 16 VAS improvement of at least 40 points for pruritus and sleep loss and 30 points for pain are presented in dupilumab‐ and placebo‐treated patients. Data are presented as the number of patients for each group. (I) Heat map representation of Dermatology Life Quality Index (DLQI). 10 questions were evaluated for each patient at week 0 and 16 of the dupilumab treatment or placebo. Rows and columns represent each DLQI question and patient, respectively. Patient numbers are reported at the bottom of the figure with patients included in the translational study highlighted in yellow. The subscore scale ranges from blue (absent) to yellow (severe). (J) DLQI evolution under dupilumab treatment or placebo. DLQI was assessed at weeks 0, 4, 8, 12 and 16. Data are presented as mean ± standard deviation. (K) Principal component analysis (PCA) approach on mTLSS, IGA, PaGA, pruritus, sleep loss and pain VAS, and DLQI at week 16 for each patient included. The plot shows the projection of data points in the first two principal components (PC1 and PC2), which represent the directions of maximum variance in the data. Each point on the plot represents all clinical scores aggregated for each patient included in the study. Patient numbers are reported above each sample. (L) Eczema Area and Severity Index evolution under dupilumab treatment or placebo. EASI was assessed at weeks 0 and 16. All patients with eczema beyond the hands (i.e., wrists, arms, feet, etc.) were included in the EASI analysis (*n* = 34 and 30 in the placebo and the dupilumab group respectively). A past or present history of AD did not influence the performance of the score. Data are presented as mean ± standard deviation. Dupilumab treated patients are labeled in dark red and placebo‐treated patients in gray. ▲ the last assessment is represented for patients who discontinued the study early (P8, P32, P46 and P47 at week 4; P9, P19, P26, P45 at week 8; P27, P42, D25 and D39 at week 12). *p < 0.05, ***p < 0.001. Wilcoxon test (B, E, F, G, J, L), two‐sided CMH‐test (H), Student's *t*‐test (C, D). AD, atopic dermatitis.

Forty‐seven patients (49.5%) were randomized to the dupilumab group, and 48 (50.5%) to the placebo group. One patient randomized to the placebo group had no baseline data recorded, did not initiate treatment, and underwent no follow‐up due to a COVID‐related disruption; this patient was therefore excluded from the study population (Figure S1). Randomization was stratified for the presence or not of a personal history of AD. Patients baseline characteristics were comparable in both groups (Table S1). At week 16, after adjusting for the stratification factor and on baseline values, the mean percentage improvement in the mTLSS in dupilumab‐treated patients was 59.8% (95% Confidence Interval, CI: 53.1–66.5) compared to 15.2% (95% CI: 4.2–26.2) in the placebo group (*p* < 0.001, Figures 2A,B, S2, and S3). These results were obtained using the LOCF (Last Observation Carried Forward) method and were consistent with sensitivity analyses performed using both Multiple Imputation and Complete‐Case Analysis. Specifically, Multiple Imputation showed a 60.9% improvement in the dupilumab group (95% CI: 54.4–67.4) versus 16.7% (95% CI: 4.3–29.1, *p* < 0.001) in the placebo group, while Complete‐Case Analysis showed a 61.5% improvement in the dupilumab group (95% CI: 55.1–67.8) versus 21.6% (95% CI: 10.7–32.5, *p* < 0.001) in the placebo group. Significant differences in the magnitude of improvement were also observed across individual mTLSS subscores, all of which showed a rapid reduction in severity, with vesicle scores demonstrating the most pronounced early response (Figure S2A–G). Importantly, the presence or not of a history of AD did not influence the clinical response (Figure 2A). Indeed, the interaction between the treatment arm and the stratification variable was tested and found to be non‐significant. In particular, dupilumab‐treated patients with baseline total IgE serum levels above 150 kUI/L (reflecting an active atopic background) and those with lower levels showed similar mean percentage improvements in the mTLSS score (57.1% (95% CI: 46.2–68.0) in patients with “high” baseline IgE levels compared to 60.1% (95% CI: 50.0–70.2) in patients with “low” baseline IgE levels). To explore potential factors associated with treatment response, we analyzed patients based on their improvement in mTLSS following dupilumab therapy, comparing those with greater than 63.64% improvement (above the median) to those with less than 63.64% improvement (below the median). This comparison included demographic characteristics, medical history, clinical parameters, and biological markers. No significant differences were identified between the two groups (Table S3). Similarly, at week 16, 53.2% (95% CI: 38.1–67.9) and 55.3% (95% CI: 40.0–69.8) of dupilumab‐treated patients achieved respectively an IGA and PaGA score of 0/1 (meaning cleared/almost cleared CHE) compared to 14.9% (95% CI: 6.2–28.3) and 10.9% (95% CI: 3.6–23.6) in the placebo group (*p* < 0.001, Figures 2C,D and S3). At week 16, mean improvements in the VAS for pruritus, sleep loss, and pain in the dupilumab group, were 41.4 points (95% CI: 33.9–48.8), 28.7 points (95% CI: 21.5–35.8), and 40.3 points (95% CI: 32.7–47.9) respectively, compared to 10.7 points (95% CI: 3.3–18.2), 5.1 points (95% CI: −2.6;12.7), and 5.0 points (95% CI: −2.6;12.6) in the placebo group (*p* < 0.001, Figure 2E–H). Regarding quality of life, the mean improvement in the DLQI in dupilumab‐treated patients was 9.3 points (95% CI: 8.1–10.5), compared to 2.5 points (95% CI: 0.8–4.2), in placebo‐treated patients (*p* < 0.001, Figure 2I,J). A clinically meaningful improvement of at least 4 points in the DLQI was achieved in 78.3% of dupilumab‐treated patients at week 16 (95% CI: 63.6–89.1) compared to 38.6% (95% CI: 24.4–54.5) in the placebo group (*p* < 0.001). Regarding the EQ5D‐5 L questionnaire evolution from baseline to week 16, 65.2% (95% CI: 49.8–78.6) of dupilumab‐treated patients showed improvement in at least one item without deterioration in any other, compared to 44.2% (95% CI: 29.1–60.1) in the placebo group (*p* = 0.050). The mean improvement in the EQ‐VAS in dupilumab‐treated patients was 15.4 points (95% CI: 9.1–21.8) compared to 7.3 in the placebo‐treated patients (95% CI: 2.6–12.0, *p* = 0.045). Considering workers (WPAI questionnaire), there was no significant change in work time missed (*p* = 0.677), in impairment while working due to health (*p* = 0.187) and in overall work impairment due to health (*p* = 0.184). Nevertheless, considering all patients (workers and non‐workers), there was a significant decrease in the mean percentage change of activity impairment due to health from baseline to week 16, in dupilumab‐treated patients (39.3% mean decrease, 95% CI: 32.7–45.9) compared to placebo (14.8% mean decrease, 95% CI: 7.5–22.1; *p* < 0.001). Of note, a period of more than 14 days (consecutive or not) out of 16 weeks during which highly potent topical corticosteroids were applied was considered potentially influential on clinical severity and thus a criterion for exclusion from the per‐protocol population. However, only 6 out of 91 patients (6.4%) met this criterion, and all were in the placebo group (*n* = 6/47, 12.8%). This strongly suggests that the positive outcomes observed in the dupilumab‐treated population were not influenced by topical corticosteroid use.

In order to visualize the extent of clinical segregation of patients depending on dupilumab or placebo intervention, the above‐mentioned clinical scores were combined in a Principal Component Analysis (PCA) approach (Figure 2K). While some placebo receivers may illustrate the “placebo effect” or topical corticosteroid rescue treatment, we could show that the variability of aggregated scores in our cohort was mainly due to the therapeutic intervention (dupilumab or placebo). Indeed, the PC1, accounting for 61% of the total variability, efficiently separates dupilumab‐ and placebo‐treated CHE patients (Figure 2K). Finally, regarding changes in eczema away from the hands, the mean improvement in EASI at week 16 in dupilumab‐treated patients was 4.2 points (95% CI: 3.6–4.9) compared to 0.4 (95% CI: −1.4; 2.2) in the placebo group (*p* < 0.001, Figure 2L).

Treatment safety was similar in both groups (Table 1). Seventy‐two patients reported 222 adverse events (AE) regardless of causality, 33 patients (70.2%, 95% CI: 55.1–82.7) in the dupilumab group compared to 39 patients (83%, 95% CI: 69.2–92.4) in the placebo group reported at least one AE. Mild to moderate ophthalmic AE were observed in 23.4% (95% CI: 12.3–38.0) of patients in the dupilumab group compared to 8.5% (95% CI: 2.4–20.4) in the placebo group (none were severe). None of the ophthalmic AE led to treatment interruption. Two serious AEs were reported in two patients, one herpes infection of the eyelid in the dupilumab group and one eczema flare requiring hospital admission for care in the placebo group.

**TABLE 1:** Collected adverse events.

	Placebo arm	Dupilumab arm	Total
	*N* = 47 (50%)	*N* = 47 (50%)	*N* = 94 (100%)
Total number of patients with at least 1 adverse event	39 (83.0%)	33 (70.2%)	72 (76.6%)
Injection site reaction	2 (4.3%)	8 (17.0%)	10 (10.6%)
Ocular adverse events	4 (8.5%)	11 (23.4%)	15 (16.0%)
Infections	12 (25.5%)	25 (53.2%)	37 (39.4%)
Ophthalmic herpes	—	1 (2.1%)	1 (1.1%)
Benign infection	11 (23.4%)	21 (44.7%)	32 (34.0%)
Skin and subcutaneous tissue disorders	19 (40.4%)	14 (29.8%)	33 (35.1%)
Eczema flare	14 (29.8%)	7 (14.9%)	21 (22.3%)
Rash	1 (2.1%)	—	1 (1.1%)
Other	7 (14.9%)	8 (17.0%)	15 (16.0%)
General symptoms	7 (14.9%)	6 (12.8%)	13 (13.8%)
Asthenia	3 (6.4%)	3 (6.4%)	6 (6.4%)
Pyrexia	1 (2.1%)	1 (2.1%)	2 (2.1%)
Other	3 (6.4%)	2 (4.3%)	5 (5.3%)
Nervous system disorders	5 (10.6%)	9 (19.1%)	14 (14.9%)
Headache	3 (6.4%)	3 (6.4%)	6 (6.4%)
Migraine	1 (2.1%)	3 (6.4%)	4 (4.3%)
Migraine with aura	—	1 (2.1%)	1 (1.1%)
Paraesthesia	—	1 (2.1%)	1 (1.1%)
Sensorimotor disorder	—	1 (2.1%)	1 (1.1%)
Syncope	1 (2.1%)	—	1 (1.1%)
Musculoskeletal and connective tissue disorders	3 (6.4%)	6 (12.8%)	9 (9.6%)
Arthralgia	3 (6.4%)	4 (8.5%)	7 (7.4%)
Sjogren's syndrome	—	2 (4.3%)	2 (2.1%)
Intervertebral disc protrusion	—	1 (2.1%)	1 (1.1%)
Gastrointestinal disorders	3 (6.4%)	6 (12.8%)	9 (9.6%)
Nausea/vomiting/Abdominal pain	1 (2.1%)	5 (10.6%)	6 (6.4%)
Other	2 (4.3%)	1 (2.1%)	3 (3.2%)
Pulmonar and ENT disorders	3 (6.4%)	3 (6.4%)	6 (6.4%)
Epistaxis	—	1 (2.1%)	1 (1.1%)
Rhinorrhoea	1 (2.1%)	1 (2.1%)	2 (2.1%)
Cough	1 (2.1%)	—	1 (1.1%)
Oropharyngeal pain	—	1 (2.1%)	1 (1.1%)
Allergic rhinitis	1 (2.1%)	—	1 (1.1%)
Lymphadenopathy	1 (2.1%)	—	1 (1.1%)
Blood disorders	—	1 (2.1%)	1 (1.1%)
Hyperleukocytosis	—	1 (2.1%)	1 (1.1%)
Lymphocytosis	—	1 (2.1%)	1 (1.1%)
Neoplasia	—	1 (2.1%)	1 (1.1%)
Basal cell carcinoma	—	1 (2.1%)	1 (1.1%)
Skin squamous cell carcinoma	—	1 (2.1%)	1 (1.1%)
Injury	—	3 (6.4%)	3 (3.2%)
Metabolism and nutrition disorders	1 (2.1%)	1 (2.1%)	2 (2.1%)
Hypercalcaemia	1 (2.1%)	—	1 (1.1%)
Vitamin C deficiency	—	1 (2.1%)	1 (1.1%)
Psychiatric disorders	1 (2.1%)	1 (2.1%)	2 (2.1%)
Anxiety	—	1 (2.1%)	1 (1.1%)
Mood alteration	1 (2.1%)	—	1 (1.1%)
Renal and urinary disorders	2 (4.3%)	—	2 (2.1%)
Dysuria	1 (2.1%)	—	1 (1.1%)
Renal colic	1 (2.1%)	—	1 (1.1%)
Reproductive system and breast disorders	1 (2.1%)	1 (2.1%)	2 (2.1%)
Dysmenorrhea	—	1 (2.1%)	1 (1.1%)
Metrorrhagia	1 (2.1%)	—	1 (1.1%)
Vascular disorders	2 (4.3%)	—	2 (2.1%)
Hot flush	1 (2.1%)	—	1 (1.1%)
Hypertension	1 (2.1%)	—	1 (1.1%)
Pregnancy, puerperium and perinatal conditions	1 (2.1%)	—	1 (1.1%)
Pregnancy	1 (2.1%)	—	1 (1.1%)

Abbreviation: ENT: ear, nose and throat.

Overall, these data strongly suggest that blocking the IL4Rα axis is effective to alleviate most of the symptoms and clinical features in our cohort patients with CHE enrolled without preselection.

### 2.3 Subhead 3: Chronic Hand Eczema Patients Share Complex Transcriptomic Core Signatures With Atopic Dermatitis and Psoriasis Patients

**FIGURE 3:**
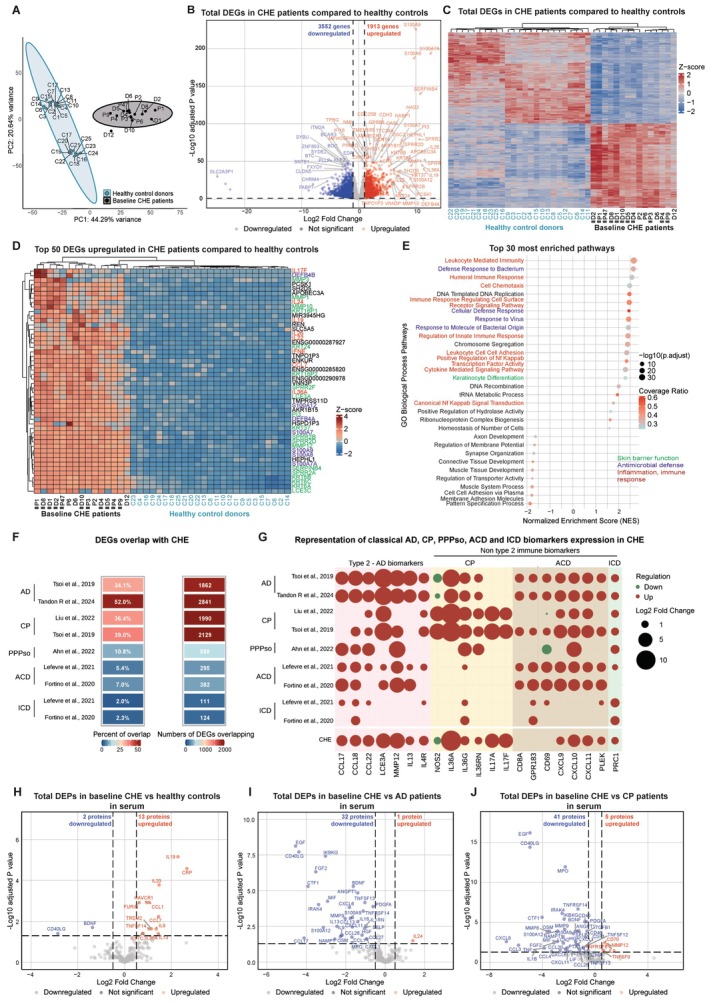
Chronic hand eczema patients share complex transcriptomic core signatures with atopic dermatitis and psoriasis patients. (A) PCA approach on the top 500 variable genes from bulk‐RNA sequencing, showing the segregation between CHE patients at baseline and healthy donor controls. Patient and control numbers are reported above each sample. Controls C1 to C15 correspond to healthy skin from the arm or abdomen, while controls C16 to C25 correspond to healthy palm skin. (B) Volcano plot representation of differentially expressed genes (DEGs) between CHE patients and healthy controls. Log_2_ fold change threshold: < −1 and > 1, adjusted *p* value threshold < 0.05. Red and blue represent up‐ and downregulated expression, respectively. (C, D) Hierarchically clustered heatmap representations of total (C) or top 50 upregulated (D) DEGs in CHE patients compared to controls. Characteristic genes involved in skin barrier functions (green), antimicrobial defense (blue) or inflammation/immune response processes (red) are highlighted. CHE patients marked with a black rectangle below their number have a history of atopic dermatitis (AD). (E) Top 30 most enriched pathways from Gene set enrichment analysis (GSEA) in CHE patients. *Y*‐axis represents ranked pathways, and *X*‐axis shows the normalized enrichment score (NES). Blue to red colors represent low to high NES, respectively. The size of the bubbles is associated with the *p* value. (D, E) Characteristic genes involved in skin barrier functions (green), antimicrobial defense (blue) or inflammation/immune response processes (red) are highlighted. (A–E) *n* = 25 controls and 15 baseline CHE patients. (F) DEGs overlap with CHE. DEGs identified in our cohort of CHE patients were compared to DEGs from patients with AD, classical psoriasis vulgaris (CP), and palmoplantar psoriasis (PPPso) based on bulk RNA‐sequencing; and from patients with allergic contact dermatitis (ACD) and irritant contact dermatitis (ICD) based on microarray data. The comparison highlights the percentage (left) and absolute number (right) of overlapping DEGs between CHE and each condition (G) Representation of classical AD, CP, ACD and ICD biomarkers expression in CHE patients. Selected AD biomarkers are *CCL17, CCL18, CCL22, LCE3A, MMP12, IL13 and IL4R*. Selected CP biomarkers are *NOS2, IL36A, IL36G, IL36RN, IL17A* and *IL17F*, selected ACD biomarkers are *CD8A, GPR183, CD69, CXCL9, CXCL10, CXCL11* and *PLEK*, ICD biomarker is *PRC1*. The size of the bubbles is related to log_2_ fold change while the color reflects downregulation or upregulation, in green and red respectively. (H–J) Volcano plot representation of differentially expressed proteins (DEPs) in CHE patients at baseline, compared to healthy controls (H), AD patients (I), and CP patients (J) in serum. DEPs were defined by a log_2_ fold change threshold of > 0.5 or < −0.5 and an adjusted *p* value < 0.05. Protein expression was quantified using the NULISAseq Inflammation Panel 250. (H) CHE baseline (*n* = 35) vs. healthy controls (*n* = 6); (I) CHE baseline (*n* = 35) vs. AD patients (*n* = 7); (J) CHE baseline (*n* = 35) vs. CP patients (*n* = 7).

To assess the extent of the molecular heterogeneity of CHE disease in our cohort, we performed whole skin bulk RNA sequencing on 15 unsorted CHE patients (*n* = 11 with AD history, *n* = 4 without AD history) sampled at baseline and 25 healthy skin controls (quality controls are described in the Materials and methods section) (Table S4). Considering our cohort of CHE patients sampled from the palm (3/15), the dorsum (9/15), and the palm/dorsum interface (3/15), we integrated palm (*n* = 10) and arm/abdomen (*n* = 15) control samples to constitute a robust representation for comparative analysis [28]. Although this dataset does not include healthy skin samples from the dorsum of the hand, recent conclusions from Quaade et al. [22] indicate that the transcriptomic profiles of skin from the dorsum of the hand are highly similar to those from other distant locations, such as the arm, with only 18 differentially expressed genes (DEGs) identified. We detected an average of 63,000 transcripts per sample (including pseudogenes and isoforms) that were further downsampled for differential analyzes of hypervariable genes to 30,000 transcripts per biopsy as previously described [29, 30]. Using a PCA approach, we observed a segregation between patients and controls into distinct clusters (Figure 3A). While control samples did not form a single homogeneous group, they remained distinct from patient samples, indicating that disease‐driven variability exceeds inter‐individual differences in healthy controls (Figures 3A–C and S4A). We next assessed DEGs between CHE patients at baseline and healthy controls. In CHE patients, we identified 3552 downregulated and 1913 upregulated DEGs, with an adjusted *p* value < 0.05, |log_2_ fold change| ≥ 1. We thus used those DEGs as the transcriptomic core signature of CHE pathology compared to steady state (Figure 3B,C). Among the most significant DEGs, we found a relatively high expression of some genes encoding typical mediators from type 1 (*GZMB, IL2RA*/CD25, *IL12RB2*, *CXCL9*, *CXCL10*, *CXCL11*, *TNFRSF9*/CD137, *TNFSF10*/TRAIL (TNF‐related apoptosis‐inducing ligand), TNFSF14), but mostly type 2 (*IL4R, IL4I1, IL13, IL13RA1, IL13RA2, CCL17*/TARC (Thymus and Activation‐Regulated Chemokine), *CCL18*, *CCL22*), and type 3 (*IL1A, IL1B, IL17A, IL17C, IL17F, IL12B (p40)*, *IL23A, IL23R, IL22, IL22RA2, IL21, IL21R, IL24, IL26, IL20, IL36A, IL36G, IL36RN, CCL20, CXCL1*) immune responses, as well as structural genes encoding proteins involved in tissue remodeling/skin homeostasis (*MMP1, MMP3, MMP10, MMP12, COL4A1, COL6A6, SERPINB3* and *4, SPINK6, PI3, LCE3C, LCE3A, SPRR2A, 2B and 2F*), stress keratins (*KRT6A‐B‐C, KRT16* and the pseudogene *KRT16P2*) and antimicrobial defense (*DEFB4A‐B, DEFB103A‐B, S100A7‐7A‐8‐9*) (Figure 3D). Using a Gene Set Enrichment Analysis (GSEA) approach, we found that most significantly enriched pathways were related to leucocyte mediated immunity, chemotaxis, innate immune response, NFκB positive regulation, cytokine mediated signaling, keratinocyte differentiation and antimicrobial defenses (Figure 3E). We next conducted an unbiased shotgun mass spectrometry analysis of whole skin proteins in an independent pool of CHE patients (*n* = 6) and healthy controls (*n* = 4) (quality controls are described in the Materials and methods section). We detected an average of 3000 proteins per sample. PCA representation confirmed that patients and controls segregated separately in two well‐defined clusters (Figure S4D). We next assessed differentially expressed proteins (DEPs) between CHE patients at baseline and healthy controls. In CHE patients, we found 94 downregulated and 52 upregulated DEPs, with an adjusted *p* value < 0.05, |log_2_ fold change| ≥ 1. Among the most significant DEPs, the relatively high expression of proteins involved in tissue remodeling/skin homeostasis (HRNR, SERPINB13, SPRR2E, KRT73, GJB2 (connexin26)) or antimicrobial peptides (S100A7A also known as psoriasin, S100A7) (Figure S4E,F) was observed. The expression of important type 3 innate inflammatory cytokines or receptors (IL36G, IL36RN, IL1RN) was upregulated in patients compared to controls without reaching statistical significance. Based on a GSEA approach adapted to proteins, the keratinization process was among the most significantly enriched pathways (Figure S4G).

To gain a better understanding of CHE molecular heterogeneity, we took advantage of publicly available transcriptomic resources from inflammatory, atopic and allergic skin diseases [31, 32, 33, 34, 35, 36] known to be directly or indirectly related to CHE pathophysiology. We initially extracted the skin transcriptomic core signature based on DEGs (adj *p* < 0.05) found in patients with AD (*n* = 2 datasets) [31, 34], psoriasis (either conventional (CP, *n* = 2 datasets) [32, 34] or palmo‐plantar psoriasis (PPPso, *n* = 1 dataset) [35]), allergic contact dermatitis (ACD, *n* = 2 datasets) and irritant contact dermatitis patients (ICD, *n* = 2 datasets) [33, 36] compared to healthy controls (Figure S4B). We then examined the potential similarities between these signatures and those of CHE patients. We observed a strong common transcriptomic signature between CHE and CP patients (ranging from 36.4% to 39%, *n* = 1990 to 2129 common DEGs) and between CHE and AD patients (ranging from 34.1% to 52%, *n* = 1862 to 2841 common DEGs) (Figures 3F and S4C). We next selected canonical biomarkers of type 1, type 2, and type 3 immune polarization [37, 38], well defined in the literature to characterize AD [31, 34], CP [32, 34], ACD and ICD [33, 36] diseases, and analyzed their expression in our cohort of CHE patients. Only DEGs that were consistently up‐ or downregulated reported in at least two published datasets of patients with AD, psoriasis (CP or PPPso), ACD, or ICD [31, 32, 33, 34, 35, 36] were retained for comparative analyses. In line with our previous findings, CHE patients display high expression of typical AD biomarkers such as *CCL17, CCL18, CCL22, IL4R, IL13, LCE3A* and *MMP12* and high expression of psoriasis biomarkers such as *PI3, IL36A, IL36G, IL36RN, IL17A and IL17F* (Figure 3G). While type 2 and type 3 immune responses appeared predominant, several biomarkers of type 1 immune activation, including *CXCL9*, *CXCL10*, and *CXCL11*, as well as the ACD‐associated marker *PLEK* and the ICD‐associated marker *PRC1*, were also found to be highly expressed in CHE patients (Figure 3G). These data demonstrate that the immune landscape in CHE skin at baseline is very heterogeneous, displaying significant overlap with transcriptomic programs characteristic of both AD and psoriasis.

As our skin transcriptomic analysis suggested a complex immune signature, we wondered if a similar cellular and molecular phenotype could be identified in the immune circulating cells and the serum of CHE patients, respectively. Using spectral flow cytometry among CD45^+^ cells, to explore lymphoid (Figures S5A,B and S6) and myeloid (Figures S5E,F and S7) populations, we found that CHE patients (*n* = 26) exhibited significantly higher number of circulating B cells and lower number of Natural Killer (NK) cells, naive CD4^+^ T cells, Th2 and Th17 cells as well as conventional dendritic cells type 1 (cDC1) and cDC2 CD5^−^ compared to healthy controls (*n* = 17) (Figure S5C,D,G). Furthermore, the inclusion of comparator groups consisting of adult patients with moderate‐to‐severe psoriasis (*n* = 6) and AD (*n* = 5), enabled cross‐disease comparisons of circulating Th cell subset distributions. Overall, circulating Th cells in CHE patients displayed less Th2/Th17 polarization compared to those in AD or psoriasis (Figure S5C,D). To get an insight into the skin immune populations of CHE patients, we used CIBERSORTx to deconvolute baseline skin transcriptomic data from baseline CHE patients and generate an estimated abundancy of immune cell composition. Consistent with an active inflammatory disease, activated memory CD4^+^ T cells, along with both resting and activated DCs, were predicted to infiltrate CHE skin at baseline (Figure S5H). Naive CD4^+^ T cells and B cells showed no significant differences. Predicted proportions of activated NK cells were found at lower levels in CHE, while resting NK cells were increased compared to healthy skin (Figure S5H).

Using shotgun proteomics on whole serum proteins, we detected an average of 500 proteins per sample (CHE patients at baseline, *n* = 7 and healthy donor controls, *n* = 4). In CHE patients' serum, we only found 14 downregulated and 4 upregulated DEPs, with an adjusted *p* value < 0.05, |log_2_ fold change| ≥ 1 (Figure S8A,B). However, disease state was the main driver of variation, as PCA analysis well‐separated CHE patients at baseline from healthy controls (Figure S8A). The highest upregulated DEP in CHE patients' serum compared to healthy donors was the properdin protein, also known as complement factor P (CFP), which is well known to amplify the immune response against a wide range of pathogens, particularly bacteria and fungi (Figure S8B). Antimicrobial peptides such as S100A7, S100A8/S100A9 (calprotectin), S100P and S100A12 were found significantly downregulated (Figure S8B). The GSEA approach performed on serum DEPs found that the most significantly enriched pathways were related to innate and adaptive immune responses (Figure S8C).

Finally, to extend our cross‐disease comparison, we analyzed serum samples using the NULISAseq 250 Proteins Immunology Panel, an attomolar‐sensitivity platform that enables high‐resolution biomarker quantification [39]. Serum from CHE patients (*n* = 35) was compared with that from healthy donors (*n* = 6), moderate‐to‐severe AD (*n* = 7), and psoriasis (*n* = 7) patients. In CHE, we identified 2 downregulated and 13 upregulated DEPs at adj. *p* < 0.05 and |log_2_FC| ≥ 0.5 (Figure 3H). Consistent with the cutaneous transcriptomic data, CHE serum showed elevated levels of type 1 marker (TNFSF14, CCL7), but was dominated by type 2 (IL9, IL13, CCL1) and type 3 proteins (IL17C, IL20, IL36A) compared to healthy controls (Figures 3H and S8D). By contrast, AD serum displayed a more pronounced type 2 profile, exemplified by higher CCL17 and IL13 levels (Figures 3I and S8D), whereas psoriasis serum was enriched for type 1 and type 3 signatures, with increased CXCL11, TNF, CCL3, CCL4, MPO, IL1B, CXCL6 and CXCL8 (Figures 3J and S8D) compared to CHE patients.

Overall, these data suggest that CHE patients share a complex transcriptomic and proteomic core signature with AD and psoriasis patients, with dysregulated genes and proteins involved in skin barrier, antimicrobial defenses, and a deeply mixed inflammation.

### 2.4 Subhead 4: Dupilumab Treatment Efficiently Restores Appropriate Skin Barrier and Immune Homeostasis Programs in Chronic Hand Eczema

**FIGURE 4:**
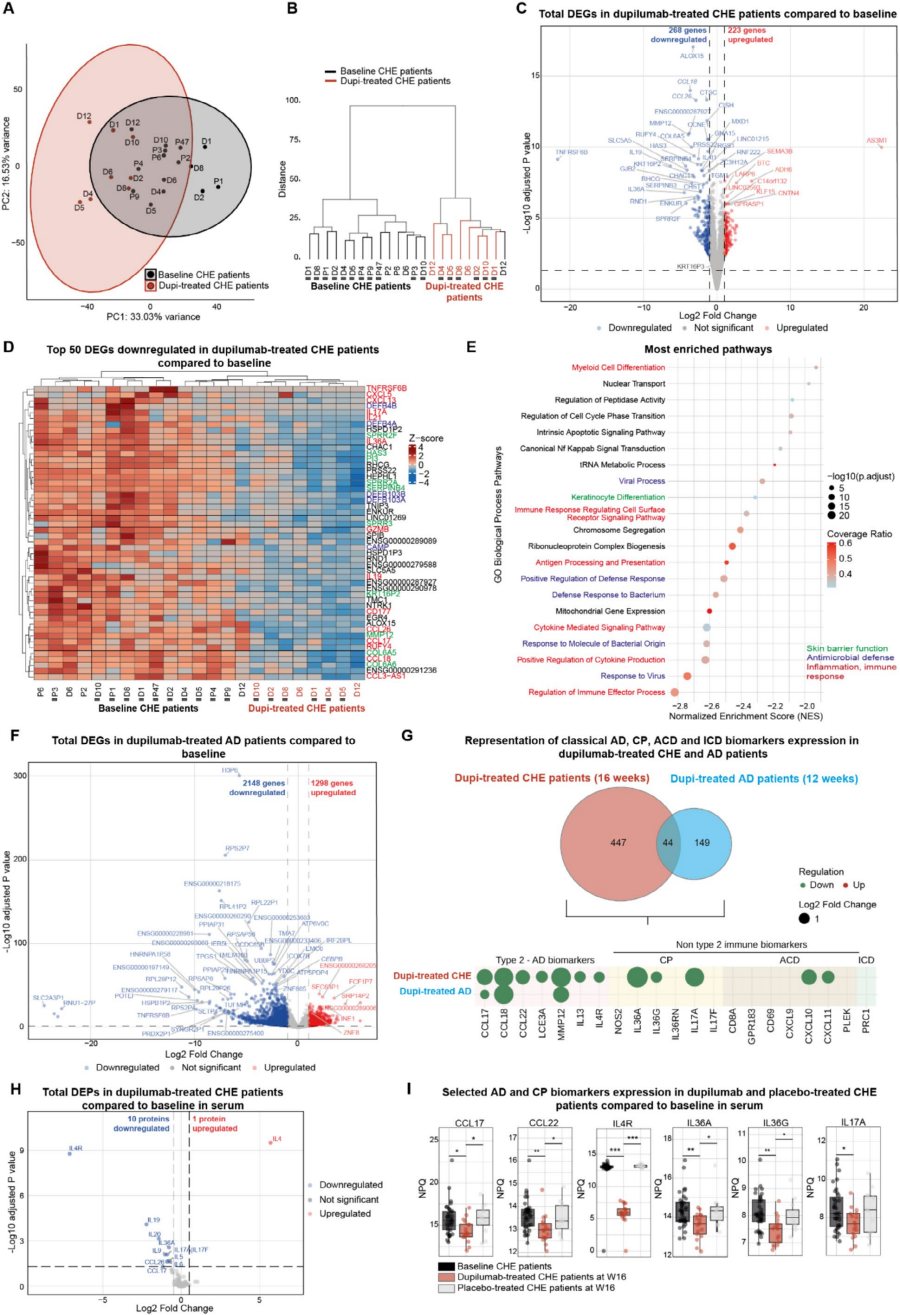
All chronic hand eczema is type 2‐driven diseases as dupilumab restores appropriate skin barrier and immune homeostasis programs. (A) PCA approach on all variable genes obtained from bulk‐RNA sequencing between baseline CHE patients and dupilumab‐treated CHE patients. Patient numbers are reported above each sample. (B) Dendrogram of the hierarchical clustering of baseline CHE patients and dupilumab‐treated CHE patients. Patient numbers are reported at the bottom of the figure. (C) Volcano plot representation of DEGs in dupilumab‐treated CHE patients compared to baseline. Log_2_ fold change threshold: < −1 and > 1, adjusted *p* value threshold < 0.05. (D) Hierarchically clustered heatmap of the top 50 downregulated DEGs in dupilumab‐treated CHE patients compared to baseline. Characteristic genes involved in skin barrier functions (green), antimicrobial defense (blue) or inflammation/immune response processes (red) are highlighted. Patient numbers are reported at the bottom of the figure. Red and blue represent up‐ and downregulated expression, respectively. CHE patients marked with a black rectangle below their number have a history of atopic dermatitis (AD). (E) Most significantly enriched pathways from GSEA in dupilumab‐treated CHE patients compared to baseline. The 21 pathways with the higher *p* value are represented. *Y*‐axis represents ranked pathways, and *X*‐axis shows NES. Blue to red colors represent low to high NES, respectively. The size of the bubbles is related to the *p* value. (D, E) Characteristic genes involved in skin barrier functions (green), antimicrobial defense (blue) or inflammation/immune response processes (red) are highlighted. (A–E) *n* = 15 baseline CHE patients and 8 dupilumab‐treated CHE patients. (F, G) Gene expression changes in the skin of dupilumab‐treated AD patients for 12 weeks compared to baseline. (F) Volcano plot representation of DEGs in CHE patients treated with dupilumab compared to AD patients treated with dupilumab. DEGs were defined using a log_2_ fold change threshold of > 1 or < −1 and an adjusted *p* value < 0.05. (G) Representation of classical biomarkers for AD, CP, ACD, and ICD in CHE or AD patients treated with dupilumab. Each sample included in the CHE or AD cohort was compared to their respective baselines. A Venn diagram identified 44 common genes, 447 CHE‐specific genes, and 149 ad‐specific genes. Selected AD biomarkers include CCL17, CCL18, CCL22, LCE3A, MMP12, IL13, and IL4R; selected psoriasis biomarkers include NOS2, IL36A, IL36G, IL36RN, IL17A, and IL17F; selected ACD biomarkers include CD8A, GPR183, CD69, CXCL9, CXCL10, CXCL11, and PLEK; the ICD biomarker is PRC1. The size of the bubbles corresponds to log_2_ fold change, while the color indicates regulation direction (green for downregulation, red for upregulation). Dupilumab‐treated CHE (*n* = 8); dupilumab‐treated AD (*n* = 22; data from Möbus et al., J Allergy Clin Immunol, 2021). (H) Volcano plot representation of DEPs in CHE patients treated with dupilumab for 16 weeks compared to their baseline in serum. Protein expression was quantified using the NULISAseq Inflammation Panel 250. DEPs were defined by a log_2_ fold change threshold of > 0.5 or < −0.5 and an adjusted *p* value < 0.05. (I) Expression of classical AD (CCL17, CCL22, IL4R) and CP biomarkers (IL36A, IL36G, IL17A) in serum of CHE patients treated with dupilumab or placebo compared to baseline. Baseline patients are shown in black, dupilumab‐treated patients in red, and placebo‐treated patients in gray. Protein levels were quantified using NULISA Protein Quantification (NPQ). *p < 0.05, **p < 0.01, ***p < 0.001. Wilcoxon test. CHE baseline (*n* = 35), dupilumab‐treated CHE (*n* = 20), placebo‐treated CHE (*n* = 15).

The randomized clinical trial demonstrated the efficacy of dupilumab in CHE patients of diverse clinical presentations and causative triggers, irrespective of their AD background. We therefore aimed to explore potential dupilumab‐associated transcriptomic changes. Among the 15 patients included in the transcriptomic analysis, 13 completed the study (i.e., *n* = 13 paired skin samples at week 16). PCA approach and hierarchical clustering showed a detectable shift of dupilumab‐treated patients compared to baseline patients (Figure 4A,B). All patients sampled included in the dupilumab group (*n* = 8) were considered responders with respect to clinical scores at week 16 (Figure 2). We found 491 significant DEGs (adjusted *p* value < 0.05, |log_2_ fold change| ≥ 1) compared to paired skin biopsies sampled before treatment (Figure 4C), with more than half of them being downregulated (*n* = 268). After dupilumab treatment, the expression of genes identified as part of the transcriptomic core signature of CHE disease was restored, including genes encoding proteins associated with type 1 (*CXCL10, CXCL11, GZMB*), type 2 (*IL4I1, CCL17, CCL18*) or type 3 immunity (*IL17A, IL22, IL23A, IL36A, IL36G, CXCL1*) (Figure 4D). Similarly, genes encoding proteins required for tissue remodeling (*SPRRR3‐2A‐2F, PI3, SERPINB4, MMP12, COL6A6*) and antimicrobial peptides (*DEFB4B‐A, DEFB103B*) were found significantly downregulated (Figure 4D). *KRT16P2*, along with classical stress keratins typically associated with epidermal hyperproliferation and barrier stress (*KRT6A*, *KRT6B*, *KRT6C*, and *KRT16*), showed normalized expression levels following dupilumab treatment in CHE patients. Notably, log_2_ fold changes for these genes decreased from a baseline range of 4.8–6.2 to −1.0 to −3.3 post‐treatment (Figure 4C,D). Overall, the main biological pathways identified at baseline in the skin transcriptome by GSEA analysis, including keratinocyte differentiation and cytokine‐mediated signaling, were significantly downregulated following dupilumab treatment (Figure 4E). Importantly, no significant changes in DEGs were observed in the placebo group (Figure S9A–C). To refine our cross‐disease comparison and evaluate treatment‐specific effects, we compared the transcriptomic changes induced by dupilumab in our CHE cohort (16‐week treatment) with the dataset reported by Möbus et al. for AD (12 weeks) [40]. Dupilumab elicited 491 DEGs in CHE and 193 DEGs in AD (adj. *p* < 0.05), 44 of which were shared between the two conditions (Figure 4F,G). These common DEGs included canonical inflammatory genes, principally type 2 markers *CCL17, CCL18, CCL11* (eotaxin‐1), along with barrier or tissue‐remodeling genes *COL6A5, COL6A6, SERPINB4* and *MMP12* (Figures 4F,G and S9D). CHE‐specific DEGs at week 16 comprised classical psoriasis markers *IL36A* and *IL17A* and type 1 chemokines *CXCL9, CXCL10* and *CXCL11* (Figures 4G and S9D). In contrast, the publicly‐available 12‐week AD dataset showed an effect largely confined to the type 2 axis (Figures 4G and S9D). In addition, genes defining the keratinocyte‐differentiation program were more downregulated in dupilumab‐treated CHE than in AD (Figure S9E). Overall, these data demonstrate that IL4Rα blockade in CHE suppresses canonical type 2 immunity‐associated genes and, at least in part, type 1 and type 3‐associated transcriptomic programs.

Among the 6 patients included in the proteomic analysis, 5 completed the study. Using a PCA approach, we confirmed a significant segregation of dupilumab‐treated patients compared to baseline (Figure S10A). All patients included in the dupilumab group (*n* = 3) were classified as responders based on clinical scores at week 16 (Figure 2). We identified 55 significantly downregulated and 61 upregulated DEPs (adjusted *p* value < 0.05, |log_2_ fold change| ≥ 1) compared to skin biopsies sampled before treatment (Figure S10B). After dupilumab treatment, the expression of key proteins previously reported to be overexpressed in dysregulated skin barrier (SPRR1A, PI3, KRT74) was significantly downregulated, while other critical components of skin homeostasis, such as FLG (filaggrin) or serpin A4 (SERPINA4), were upregulated (Figure S10B–D).

At the protein level, only 2 out of the 3 placebo‐treated patients sampled at baseline completed the study; therefore, we could not investigate with enough statistical confidence DEPs between the two groups. However, based on the PCA graph (Figure S10E), we anticipate that no biologically significant differences would distinguish the placebo‐treated patients from those at baseline. In line with our findings in immune circulating populations and shotgun proteomic in the serum of CHE patients at baseline, we explored the possibility of restoring normal levels of B cells, NK cells, and DCs after treatment. No major change was observed in all tested immune cell populations (Figure S11A–F) both in the dupilumab and the placebo groups. Based on immune cell composition estimates in the skin using CIBERSORTx, DCs and memory CD4^+^ T cells were predicted to be less abundant in CHE patients treated with dupilumab compared to those receiving placebo (Figure S11G). While no major change was observed in untargeted serum proteins following dupilumab or placebo treatment (Figure S12A–D), the use of the NULISA detection system revealed that serum levels of many proteins upregulated at baseline were largely normalized following dupilumab treatment, with 10 significantly downregulated DEPs identified, compared to none in the placebo group (Figures 4H,I and S12F). These included canonical type 2 and type 3 immune mediators such as IL4R, IL5, CCL17, CCL26, IL6, IL17A/F, and IL36A. Only one DEP was found to be significantly upregulated in dupilumab‐treated patients, namely IL4, likely reflecting an increase in the unbound fraction of IL4 secondary to IL4Rα blockade (Figures 4H,I and S12F).

Taken together, these results indicate that, compared to placebo control, short‐term dupilumab treatment significantly regenerates appropriate biological programs related to skin barrier and immune homeostasis. These findings suggest that IL4Rα signaling may be a critical regulator of CHE pathological features and inflammatory signatures, regardless of AD background or environmental triggers.

## 3 Discussion

Our findings reveal that chronic hand eczema patients exhibit a mixed immune signature sharing features of both atopic dermatitis and psoriasis, along with pronounced skin barrier defects. Remarkably, short‐term dupilumab treatment significantly improved clinical signs and symptoms of the disease and restored appropriate biomarkers of skin barrier and immunity in unselected patients.

CHE is a highly heterogeneous condition, encompassing both atopic and non‐atopic origins as well as diverse clinical manifestations, all of which were represented among our cohort and associated with significant functional disabilities and pain. While our CHE patients may appear atopy‐biased due to the high prevalence of atopic background, no inclusion criteria regarding AD history or atopic predisposition were applied across the four participating centers. We believe that the enrolled population, with a mean CHE duration of almost 9 years, reflects real‐life patients requiring systemic treatment due to disease chronicity. Such individuals are indeed more likely to exhibit an atopic profile compared to those with milder or shorter‐duration CHE. It is also important to note that both AD history and atopic comorbidities were self‐reported, which may have contributed to the elevated atopic rates observed in our cohort.

While patients could have been empirically categorized based on both etiology (e.g., ICD, ACD, AD) and clinical presentation (e.g., vesicular, fissured, and hyperkeratotic CHE) [16, 17], we here chose to unambiguously study the baseline molecular signature and therapeutic response across the entire cohort of unclassified patients. Indeed, there is no consistent correlation between etiology and clinical presentation, and the vast majority of CHE patients cannot be easily classified in a unique subtype (e.g., ICD, ACD, AD) despite thorough interrogation, clinical examination and allergy testing [16, 19, 41, 42].

The skin of CHE patients at baseline showed a pronounced mixed immune signature indicative of a concomitant type 2 and type 1/3 inflammation, along with a severely disrupted skin barrier. Genes typically associated with type 2 immune responses (e.g., *IL4R, IL13, CCL17, CCL18*), as observed in AD, were strongly expressed in CHE skin. A strong psoriasis‐like component of the disease was also evident, characterized by the marked expression of type 3 immunity‐related genes such as *IL17A* and *IL17F*, alongside markers from the IL36 family, commonly linked to pustular psoriasis [34]. While some classical psoriasis biomarkers were shared between CHE and AD, others, such as *IL17A*, and *IL17F*, seemed more specific to CHE and psoriasis cohorts. Type 1‐associated transcripts (e.g., *CXCL10, GZMB*) were also detected, albeit to a lesser extent. Notably, the key immune biomarkers identified at the transcriptomic level in the skin were also detected in the serum of patients. The severely disrupted skin barrier, typically seen in patients with chronic fissures and hyperkeratosis, was reflected at the transcriptomic and proteomic levels by differentially expressed genes and proteins involved in skin remodeling and keratinocyte differentiation. These included Matrix Metalloproteinases (MMPs), Collagens (COLs), Serine Protease Inhibitors (SERPINs), other protease inhibitors like SPINK, Small Proline‐Rich Proteins (SPRRs), and stress keratins—molecules typically associated with hyperproliferative and stress‐induced responses in the epidermis—all indicative of excessive tissue damage and the skin's attempt to remodel and repair itself. Overall, we found that the clinical and etiological diversity of CHE is reflected in its mixed AD/psoriasis immune signature, alongside a severely compromised skin barrier.

Effective treatment options for patients with moderate to severe CHE are limited [6, 17, 41]. Dupilumab, originally licensed for the treatment of moderate‐to‐severe AD [8], has been preliminarily tested in randomized clinical trials for its efficacy in CHE [26, 27]. The first trial focused on a highly selective group of patients with AD affecting the hands and feet [26], while the second targeted patients with subtypes of recurrent vesicular or chronic fissured CHE [27]. In both studies, dupilumab showed significant efficacy compared to placebo at week 16 [26, 27], yet the patient populations studied did not represent the full spectrum of CHE heterogeneity. Our study extends our knowledge of CHE beyond these findings, demonstrating that short‐term dupilumab treatment significantly improves clinical severity and quality of life in patients with all forms of CHE. Importantly, this is achieved while largely restoring appropriate transcriptomic and proteomic programs related to skin barrier integrity and immune homeostasis.

Despite the wide variability in clinical presentation, origins and baseline mixed immune signature, the clinical response to IL4Rα blockade with dupilumab was remarkably consistent across the entire cohort, regardless of AD history and, more generally, irrespective of current atopic imprinting as reflected by total IgE serum levels. The restoration of molecular profiles related to skin barrier function and immune regulation supports the idea that targeting IL4Rα signaling may be an effective therapeutic strategy to reverse both clinical and molecular manifestations of the disease. However, long‐term follow‐up of patients is warranted to draw more robust conclusions. This uniform response suggests that type 2 immunity may participate in orchestrating, or sustaining, the coupled type 1 and type 3 inflammatory pathways in CHE. Obviously, CHE should not be classified as a strictly ‘type 2’ disease; however, it may be characterized as a heterogeneous immune condition with a certain degree of IL‐4Rα dependence, at least in our cohort of patients. IL4Rα is broadly expressed on naive and early‐activated T cells, regardless of their eventual polarization. Upon initial TCR activation, CD4^+^ T cells transiently upregulate IL4Rα, making them responsive to IL4 signaling [43, 44, 45, 46]. This early, non‐selective expression of IL4Rα, combined with its presence on other cells such as peripheral sensory neurons—which are known to interact with immune cells, particularly in inflammatory skin diseases—may explain, at least in part, why IL4Rα blockade results in broader immunomodulatory effects beyond type 2 pathways [47, 48, 49, 50]. This mechanism could potentially interfere with T cell activation and differentiation in a more global manner. Altogether, our findings support the use of treatments targeting the type 2 immune response, as well as therapies with broader immunosuppressive effects such as topical or systemic JAK inhibitors, for all patients with CHE, regardless of their clinical subtype or environmental triggers. Notably, no clinical trial has yet evaluated the strategy of targeting type 1 or type 3 immunity in CHE patients. Importantly, controlling exposure to hand barrier–damaging substances (or allergens) should always be considered an essential component of the therapeutic strategy.

Our results build upon preliminary analyses investigating the molecular profile of CHE [23, 24, 25, 51], while those studies did not explore the associated therapeutic approach. Quaade et al. [22] recently showed that the lesional palmar transcriptome of CHE is largely shared between patients with and without AD, as well as across different etiologies, supporting the existence of a common molecular CHE endotype. In line with our findings, these data also revealed a heterogeneous inflammatory response, predominantly characterized by Th1 activation, followed by notable Th2 activity and Th17 involvement [22]. Key upstream regulators shared across CHE subtypes included type 1‐associated molecules (*IFNG, TNF, STAT1, IL2*), type 2‐associated molecules (*IL4*), type 3‐associated molecules (*IL17A*, *IL22*, *IL1B*) [22]. Another study reported that upregulated serum proteins in patients with severe CHE, classified under the ACD subtype without concurrent AD, included type 2 inflammatory markers like *CCL17* and *CCL13*, along with type 1 inflammatory markers [18]. To date, no robust biomarkers have been identified that reliably classify CHE subtypes. Some authors detected inflammatory markers in the serum of CHE patients using the targeted Olink Proseek Multiplex Assay [18]. In our study, the analysis of blood immune cell populations by spectral flow cytometry or global shotgun proteomics in the serum revealed only minimal differences between CHE patients at baseline and healthy controls, with no significant changes following dupilumab treatment. Based on these findings, we could speculate that blood immunophenotyping or serum untargeted proteome analysis might not be a sensitive enough readout for monitoring a predominantly locally‐restricted condition like CHE. Conversely, the high sensitivity of NULISAseq technology enabled the detection and monitoring of key biomarkers, including type 1, type 2, and type 3 immunity‐related cytokines and chemokines, in patient serum. These serum proteins showed strong concordance with gene expression levels in the skin and were significantly associated with treatment response. Of note, the skin proteomic analysis was constrained by the limited sample size due to the optional nature of biopsies and the prioritization of tissue for transcriptomic profiling. Although these data do not allow for definitive conclusions, we believe they offer preliminary yet meaningful insights when integrated with the comprehensive gene, protein, and cellular characterization performed in both skin and blood of CHE patients.

From a clinical view, our findings provide a perspective similar to the one obtained in patients suffering from prurigo nodularis, a chronic inflammatory skin disease characterized by intensely pruritic nodules, which may be associated with a history of atopy or current atopic comorbidities, such as AD in up to 47% of patients [52, 53, 54]. In two phase 3 clinical trials evaluating dupilumab versus placebo at week 24, efficacy outcomes were similar between atopic and non‐atopic patients [55]. Although more than 50% of enrolled patients did not have an atopic background, dupilumab efficacy was consistent regardless of atopic or non‐atopic status [55]. In line with this, the skin transcriptome of patients with palmoplantar pustulosis has shown greater overlap with that of AD than with classical psoriasis [56], and real‐world data suggest efficacy of dupilumab in this setting [57]. Such mixed profiles, seen both in our data and in the literature, suggest that immune signatures are rarely confined to one specific type of inflammation.

Taken together, these data reveal the mixed immune nature of CHE and support IL‐4Rα blockade as a valuable therapeutic approach in unselected moderate‐to‐severe patients.

## Author Contributions

Conceptualization: Marie Tauber, Emilie Bérard, Carle Paul, Nicolas Gaudenzio. Methodology: Marie Tauber, Carle Paul, Nicolas Gaudenzio, Emilie Bérard, Jonathan Rioual, Lilian Basso, Marine A. Lefevre. Investigation: Marie Tauber, Perrine Gery, Vanina Lenief, Hugo Rimet, Rüçhan Ekren, Nicolas Gaudenzio, Marc Vocanson for the experiments; Marie Tauber, Manon Boussier, Aurélie Du‐Thanh, Nadia Raison‐Peyron, Fatma Jendoubi, Amel Bouznad, Maria P. Konstantinou, Cristina Bulai Livideanu, Carle Paul, Julien Seneschal, Brigitte Milpied, Audrey Nosbaum, Florence Hacard, Louise Jaulent for the clinical trial. Visualization: Perrine Gery, Hugo Rimet, Rüçhan Ekren, Vanina Lenief, Marie Tauber. Funding acquisition: Marie Tauber, Carle Paul, Marc Vocanson, Nicolas Gaudenzio. Project administration: Marie Tauber, Carle Paul, Nicolas Gaudenzio. Supervision: Marie Tauber, Nicolas Gaudenzio. Writing – original draft: Perrine Gery, Marie Tauber, Nicolas Gaudenzio, Vanina Lenief, Hugo Rimet, Rüçhan Ekren. Writing – review and editing: all authors. The study design, manuscript writing, ethical submission, clinical trial conduct, and data analysis were all carried out independently of Sanofi. As this was an investigator‐initiated study funded by Sanofi (for the RCT only, with no funding provided for the translational research), Sanofi US had access to the study protocol but did not participate in the study design, data collection, or interpretation. All steps were overseen and conducted exclusively by academic physicians and researchers.

## Funding

This work was supported by Sanofi US ISS, Acteria Fundation, Agence Nationale de la Recherche, European Research Council.

## Conflicts of Interest

Perrine Gery, Rüçhan Ekren, and Emilie Bérard have no competing interests. Aurélie Du‐Thanh: investigator, consultant or speaker for AbbVie, Sanofi, Pfizer, Leo Pharma and Lilly. Hugo Rimet, Fatma Jendoubi, and Vanina Lenief have no competing interests. Nadia Raison‐Peyron: arrangement fees from Sanofi. Amel Bouznad: consultant for UCB, Janssen, Amgen, Abbvie, Novartis, Sanofi. Maria P. Konstantinou, Jonathan Rioual, Lilian Basso, Juliette Mouiren, Manon Boussier, Brigitte Milpied, Cristina Bulai Livideanu, Louise Jaulent have no competing interests. Florence Hacard: investigator, consultant or speaker for Sanofi, Leo Pharma, Galderma, Abbvie, Almirall. Marine A. Lefevre: investigator, consultant or speaker for Sanofi, Leo Pharma, Abbvie. Audrey Nosbaum: investigator, consultant or speaker for Pfizer, AbbVie, Eli Lilly, Galderma, Leo Pharma, Novartis, Sanofi, Incyte. Julien Seneschal: consultant, investigator for AbbVie, Almirall, Bristol Myers Squibb, Eli Lilly, Leo Pharma, Pfizer, Pierre Fabre, Sanofi. Marc Vocanson have no competing interests. Carle Paul: investigator, consultant for Amgen, Abbvie, Bristol Myer Squibb, Boehringer, Celgene, Eli Lilly, Novartis, Janssen, Pierre Fabre, Pfizer, Leo Pharma. Nicolas Gaudenzio: consultant for Sanofi. Marie Tauber: Investigator, consultant or speaker for Sanofi, Leo Pharma, Galderma, Abbvie, Almirall, Medac, Lilly, Pfizer.

## Supporting information


**Table S1:** Baseline patients characteristics.
**Table S2:** Baseline CHE disease characteristics.
**Table S3:** Analysis of factors influencing dupilumab efficacy based on primary endpoint response.
**Table S4:** Overview of patients included in the molecular phenotyping study.
**Figure S1:** Flow chart of the clinical study.
**Figure S2:** Evolution of Modified Total Lesion Symptom Score (mTLSS) subscores and Eczema Area and Severity Index (EASI) lower extremities score under dupilumab or placebo treatment from W0 to W16.
**Figure S3:** Representative pictures of CHE evolution in four placebo‐treated and four dupilumab‐treated patients from baseline to week 16.
**Figure S4:** Baseline whole skin proteomic signature in CHE patients compared to healthy controls was restricted to a strongly compromised skin barrier.
**Figure S5:** Baseline CHE patients exhibited fewer alterations in circulating immune cells compared to AD or psoriasis controls.
**Figure S6:** Gating strategy for lymphoid cells analysis by spectral flow cytometry.
**Figure S7:** Gating strategy for myeloid cells analysis by spectral flow cytometry.
**Figure S8:** At baseline, CHE patients exhibited a mixed immune activation profile in serum compared to healthy, AD, and psoriasis controls.
**Figure S9:** DEGs comparison between dupilumab‐treated CHE and AD patients in the skin.
**Figure S10:** Skin barrier defect proteomic signature was restored after 16 weeks‐dupilumab treatment compared to placebo in CHE patients.
**Figure S11:** No major change was observed in circulating immune cells in dupilumab‐treated patients compared to placebo at week 16 in CHE patients.
**Figure S12:** Decreased type 2 and type 3 immune responses observed in dupilumab‐treated CHE patients in serum.

## Data Availability

The data that support the findings of this study are openly available in Zenodo at https://doi.org/10.5281/zenodo.13918363, reference number GSE279422.
