# Supplementary material for: Integrated Clinical Trial and Molecular Profiling Reveals Immune Drivers of Chronic Hand Eczema
Source: Allergy. 2026 Feb 18;81(8):2815–32. doi: 10.1111/all.70263 (PMC13465994; doi:10.1111/all.70263)
Supplement: Supplementary file 1 [file ALL-81-2815-s001.docx]

**List of Supplementary Materials**

**Materials and Methods**

**Supplementary Table 1.** Baseline patients characteristics.

**Supplementary Table 2.** Baseline CHE disease characteristics.

**Supplementary Table 3.** Analysis of factors influencing dupilumab efficacy based on primary endpoint response.

**Supplementary Table 4.** Overview of patients included in the molecular phenotyping study.

**Supplementary Figure 1.** Flow chart of the clinical study.

**Supplementary Figure 2.** Evolution of Modified Total Lesion Symptom Score (mTLSS) subscores and Eczema Area and Severity Index (EASI) lower extremities score under dupilumab or placebo treatment from W0 to W16.

**Supplementary Figure 3.** Representative pictures of CHE evolution in four placebo-treated and four dupilumab-treated patients from baseline to week 16.

**Supplementary Figure 4.** Baseline whole skin proteomic signature in CHE patients compared to healthy controls was restricted to a strongly compromised skin barrier.

**Supplementary Figure 5.** Baseline CHE patients exhibited fewer alterations in circulating immune cells compared to AD or psoriasis controls.

**Supplementary Figure 6.** Gating strategy for lymphoid cells analysis by spectral flow cytometry.

**Supplementary Figure 7.** Gating strategy for myeloid cells analysis by spectral flow cytometry.

**Supplementary Figure 8.** At baseline, CHE patients exhibited a mixed immune activation profile in serum compared to healthy, AD, and psoriasis controls.

**Supplementary Figure 9.** DEGs comparison between dupilumab-treated CHE and AD patients in the skin.

**Supplementary Figure 10.** Skin barrier defect proteomic signature was restored after 16 weeks-dupilumab treatment compared to placebo in CHE patients.

**Supplementary Figure 11.** No major change was observed in circulating immune cells in dupilumab-treated patients compared to placebo at week 16 in CHE patients.

**Supplementary Figure 12.** Decreased type 2 and type 3 immune responses observed in dupilumab-treated CHE patients in serum.

**MATERIALS AND METHODS**

**1. Clinical Study Design and Ethics**

**Study Design**

This study was a Phase 2b, multicenter, double-blind, French academic-sponsored clinical trial ([NCT03861455](https://clinicaltrials.gov/study/NCT03861455)). We enrolled adults with moderate-to-severe CHE, defined by an Investigator Global Assessment (IGA) score of 3 or 4 (on a scale of 0 to 4). Inclusion required disease persistence for at least six months and resistance or intolerance to potent/very potent topical corticosteroids.

**Exclusion Criteria**

Patients were excluded if they had utilized: (1) topical corticosteroids or topical calcineurin inhibitors within one week prior to baseline, (2) oral immunosuppressants (including cyclosporine, methotrexate, azathioprine, mycophenolate mofetil), alitretinoin, or phototherapy within four weeks prior to the baseline visit, or (3) investigational drugs within eight weeks (or five half-lives) prior to baseline.

**Randomization and Treatment**

Participants were randomized 1:1 to receive subcutaneous dupilumab (600mg initially, then 300 mg every other week) or placebo and stratified by history of AD. Rescue therapy with potent topical corticosteroids was allowed for a maximum of 14 days; however, usage exceeding 2 weeks (consecutive or cumulative) was considered potentially influential on the evolution of clinical severity.

**Data Collection**

Epidemiological data, profession, past medical history, past medical treatment for CHE, atopic background, proven allergic contact dermatitis, exposure to irritants at home, leisure or work, and worsening of CHE at work were collected at baseline. Full hands and body examination were detailed at baseline. Of note, plantar lesions were not evaluated in this study.

**Objective and Endpoints**

The primary objective of the study was to demonstrate that dupilumab was more effective than placebo for treating patients with moderate to severe CHE resistant or intolerant to potent or highly potent topical steroids.

- **Primary Endpoint:** The percentage improvement in the modified Total Lesion Symptom Score (mTLSS) at Week 16 (W16). The mTLSS combines six morphological criteria (erythema, scaling, lichenification/hyperkeratosis, vesicles, edema and fissures) with pruritus and pain intensity.
- **Secondary Endpoints:** The percentage of patients achieving an IGA or PaGA score of 0/1 (meaning cleared or almost cleared CHE), the evolution of visual analogical scales (VAS) for pruritus, sleep loss, and pain, as well as quality of life scores including the DLQI (Dermatology Life Quality Index) and EQ-5D and the WPAI (Work Productivity and Activity Impairment) score were evaluated at W16. All performed scores (excluding WPAI) were also assessed at W4, W8 and W12. Tolerance data were collected prospectively.

**Study approval**

The study was approved by the French national ethics committee (RC31/18/0269, agreement n°2018T1-17) and registered on clinicaltrials.gov under NCT03861455. All patients provided written informed consent prior to participation in the clinical study, including blood and skin sampling as well as photographs.

**Inclusion & Ethics Statement**

This clinical trial and translational study were conducted in full compliance with ethical standards and regulatory requirements. Ethical approval was obtained from the relevant institutional review boards, and all participants provided written informed consent prior to inclusion. We ensured equitable inclusion across gender, age, ethnicity, and socio-economic background, and took steps to minimize any form of bias or discrimination throughout the study design, recruitment, data collection, analysis, and reporting. The study adhered to the principles of the Declaration of Helsinki and Good Clinical Practice (GCP) guidelines.

**2. Sample Collection and Processing**

**Skin Samples**

A baseline 4-mm biopsy was taken on lesional skin from the back of the hand whenever possible. When the back of the hand was unaffected at baseline, biopsy was taken on lesional skin from the interface of the back and palm of the hand. When the interface zone was unaffected too, biopsy was taken in the lesional palm. Biopsies were repeated at W16, 1 to 2 cm far from the baseline sampling site. There was no specific selection process for assigning patients to molecular analyses. Biopsies were optional and not financially compensated, they were performed independently of treatment allocation (placebo or dupilumab) and without prior knowledge of treatment arm. No criteria other than the patient’s written consent were applied.

- *Healthy Controls (Transcriptomics):* Obtained via GENOSKIN® from surgical residues of skin plastic surgeries (abdomen and arms, n=15). The data set of in-house controls were combined with a published dataset from Wiedemann, J., et al., (28), which includes healthy skin controls from the palm of the hands (n=10). The dataset was downloaded, reanalyzed and integrated into our bulk RNA-seq analysis alongside the abdomen and arm control samples we had previously obtained from healthy donors.
- *Healthy Controls (Proteomics):* Obtained from the dorsum of the hands (n=3). In-house controls and patients were age- and sex-matched.

**Blood Samples**

- **PBMC isolation.** Blood was diluted 1:2 with 1X PBS. PBMCs were separated by Ficol gradient centrifugation (Ficoll-Paque PLUS, Thermo Fisher Scientific©). Cells were resuspended with 500 μl of Fetal Calf Serum (SVF- CVFSVF00-01 Eurobio) and repartitioned into 1.5 mL cryotubes. Next, 500 μl of dimethil sulfoxide (DMSO-D2650-5X5ML Sigma Life Science) was gradually added to the suspension to give a final concentration of 10%. Cryotubes containing PBMCs were frozen at -80°C.
- **Serum preparation.** Serum from healthy donors and patients were isolated from 10 mL whole-blood samples using centrifugation at 2000 g for 10 min at 4°C. Serum samples were stored in Cryotubes at -80°C.

For blood samples controls, we used adult healthy donor controls from the Etablissement Français du Sang (n=6), adult patients with moderate-to-severe (e. g., IGA ranging from 2 to 4) psoriasis (n=7) and moderate-to-severe (e. g., IGA ranging from 2 to 4) AD (n=7), enabling cross-disease comparisons.

**3. Targeted Immunological Profiling**

**Spectral Flow Cytometry**

PBMC obtained were analyzed through flow cytometric experiments. Samples were transferred to a 37°C water bath to defrost, then were washed with RPMIc. Cell viability was assessed using trypan blue exclusion. Cell suspensions were stained with fluorescently labeled monoclonal antibodies (mAbs), and fixable viability staining was performed to exclude dead cells from the analysis (ViaKrome 808, Beckman). The stained cells were analyzed using the Aurora flow cytometer (Cytek® 5L 16UV-16V-14B-10YG-8R). Sample compensation was done on SpectroFlo® software. For gate-driven analyses, flow cytometry data was analyzed using FlowJo™ (Tree Star, Ashland, OR, United States) and the results were plotted using GraphPad Prism (GraphPad Software, La Jolla, CA).

- **mAbs used for FACS staining.** Anti-CD8 (RPA-T8), anti-CD7 (M-T701), anti-CD127 (HIL-7R-M21), anti-TCRab (IP26), anti-CD14 (M5E2), anti-CD11c (3.9), anti-CD1a (SK9), anti-CD34 (8G12), anti-CD4 (RPA-T4), anti-CD56 (TULY56), anti-CD19 (SJ25C1), anti-CLA (HECA-452), anti-CD45 (HI30), anti-TCRgd (B1), anti-CD94 (131412), anti-CD45RA (HI100), anti-CCR4 (L291H4), anti-CCR6 (AFKJS-9), anti-CXCR3 (TWAJ), anti-CD25 (M-A251), anti-CCR10 (314305), anti-CD294 (REA598), anti-PD1 (EH12.1), anti-CD71 (L01.1), anti-CD117 (104D2), anti-CCR7 (G043H7), anti-CD154 (REA 238), anti-CD137 (C65-485), anti-CD38 (HB7), anti-CD45RO (UCHL1), anti-CD57 (HNK-1), anti-Ki-67 (367-5698-82), anti-CD16 (3G8), anti-CX3CR1 (2A9-1), anti-CD141 (1A4), anti-CD14 (M5E2), anti-CD123 (7G3), anti-CD272 (J168-540), anti-CD163 (GHI/61), anti-CD80 (MEM-233), anti-CD1c (AD5-8E7), anti-CD11c (B-Ly6), anti-HLA-DR (G46-6), anti-CD3 (SK7), anti-CD4 (SK3), anti-CD8 (RPAT8), anti-CD19 (LT19), anti-CD86 (FUN-1), anti-CD5 (UCHT2), anti-CD303 (201A), anti-CD2 (RPA-2.10), anti-CD26 (M-A261), anti-CD33 (P67.6), anti-FcERI (AER-37), anti-IGSF4A (41N3D3), anti-CD88 (D53-1473), anti-CD89 (A59). mAbs were purchased from BD Biosciences (Le Pont de Claix, France), Miltenyi Biotech (Bergish Gladbach, Germany), eBioscience (now Thermo Fisher Scientific, Villebon-sur-Yvette, France), BioLegend, and R&D Systems (Lille, France).
- **OMIQ Analysis.** For multiparametric analysis, dimensional reduction and clustering were done using OMIQ data analysis software (OMIQ, Inc. Santa Clara, CA). Prior to analysis, FlowJo was used to clean the data by removing aggregates, dead cells, and debris. All CD45^+^ cells from all samples were selected for subsequent analysis on the OMIQ platform.

**NULISAseq™ Inflammation Panel 250 on serum.**

Eighty-six serum samples were profiled on a single plate using the NULISAseq™ Inflammation Panel 250 on the Alamar ARGO™ HT #B27 platform, targeting 250 inflammatory cytokines and chemokines. Quality controls comprised sample controls (SC, n=3), inter-plate controls (IPC, n=3) and negative controls (NC, n=4). Raw counts were first normalized intra-well to the internal control (IC) and then inter-plate by dividing by the IPC target median and rescaling (×10⁴). After adding 1, data were log₂-transformed to yield NPQ (NULISA Protein Quantification) units.

Differential abundance was assessed with unpaired or paired t-tests, or Wilcoxon tests, as appropriate. P values were corrected for multiple testing using the Benjamini–Hochberg procedure; proteins were deemed significantly regulated when FDR < 0.05 and |log₂FC| ≥ 0.5. Comparisons included: V1 (Baseline) patients versus controls, V1 versus atopic dermatitis (AD) or psoriasis subsets, and V9 versus V1 in the dupilumab and placebo arms (paired and unpaired analyses). Results were visualized with volcano plots and boxplots annotated with adjusted P values.

**4. Shotgun Proteomics: Workflow and Data Analysis Algorithm**

**Sample Preparation**

**Serum samples treatment.** Low-abundant proteins of interest were enriched onto paramagnetic beads with the ENRICH-iST kit used with 20 μL of each serum sample. The proteins recovered by the beads were then digested with a mix of Trypsin and LysC, following the PreOmics instructions for the use of iST-BCT kit (PreOmics, GmbH). Peptides were purified using mixed mode reverse phase cation exchanger SPE column (PreOmics GmbH), eluted, dried, and solubilized in 100 μL of 3% acetonitrile 0.1% formic acid aqueous solution. Then, peptide concentration in each sample was determined using the BCA method.

**Skin samples treatment.** All samples were transferred directly into clean 1.5 mL Eppendorf tubes. Each sample was heated in lysis buffer (2% SDS; 100 mM HEPES pH8.5; 10 mM TCEP; 30 mM Chloroacetamide; 1 mM EDTA and Antiprotease cOmplete) and then successively ground with UFO beads and sonicated by micro cavitation (Bioruptor Pico, Diagenode) with glass beads (Diagenode protein extraction beads). The beads were rinsed with 100 μL HEPES buffer before a centrifugation step. The protein concentration of the supernatant was determined by a BCA method compatible with reducing agents. 100 μg of proteins were precipitated with SP3 beads (PreOmics, GmbH) and digested with a mix of Trypsin and LysC, following the PreOmics instructions for the use of iST-BCT kit (PreOmics, GmbH). Peptides were purified using mixed mode reverse phase cation exchanger SPE column (PreOmics GmbH), eluted, dried and solubilized in 100 μL of 3% acetonitrile 0.1% formic acid aqueous solution. Then, peptide concentration in each sample was determined using the BCA method.

**LC-MS/MS** **Data Acquisition**

Peptides (250 ng/sample) were injected into a Vanquish Neo™ UHPLC system (Thermo Scientific) coupled to an Exploris 480 mass spectrometer (Thermo Scientific).

- **Chromatography:** PepMap100 C18 (75 μm x 50 cm, 2 μm material) column, 90 minutes gradient (2.5 % to 45% acetonitrile), flow rate300 nl/min, after a 3 minutes trapping step on precolumn.
- **MS:** MS/MS scans on most intense ions of each MS1 survey scan (2 seconds cycle time).
- **Abundance Retrieval:** Protein abundances of both blood and serum samples were retrieved via Proteome Discoverer (v3.0), Thermo Scientific).

**Proteomics Data Analysis Algorithm**

Data processing was performed using DEP (Differential Enrichment analysis of Proteomics data) R package (X. Zhang, A. H. Smits, G. B. van Tilburg, H. Ovaa, W. Huber, M. Vermeulen, Proteome-wide identification of ubiquitin interactions using UbIA-MS. *Nat. Protoc.* **13**, 530–550 (2018)) following this stepwise algorithm.

- **Step 3.1: Parsing and Filtering.** Protein abundances were filtered to retain high/medium confidence identifications (based on FDR) and to remove contaminant proteins. Protein identifiers were standardized to gene symbols and accession IDs. Sample metadata were parsed from file names to assign experimental conditions (baseline CHE patients, healthy donor controls etc.), to rename columns accordingly, and to group replicates. Redundant protein entries were collapsed by generating unique identifiers, and proteins not quantified in every replicate of at least one condition were removed.
- **Step 3.2: Normalization.** Remaining intensities were normalized using Variance‑ Stabilizing Normalization (VSN).
- **Step 3.3: Imputation.** Missing values, which were considered Missing Not At Random (MNAR), were imputed using the “MinProb” method, drawing values from a Gaussian distribution at a minimal probability level.
- **Step 3.4: Dimensionality Reduction.** To evaluate global variability, principal component analysis (PCA) was applied without scaling to the 500 most variable biopsy proteins, and to the 350 most variable serum proteins using the “prcomp” function in R. Results were visualized with ellipses that were drawn as polygons representing a 0.99 confidence level and color coding to indicate experimental conditions.
- **Step 3.5: Differential Expression**. Differential protein expression was assessed by fitting a linear model (terms: condition + replicate). Significance threshold: Adjusted p-value < 0.05 and |log₂FC| > 1. Visualization: Volcano plots and heatmaps.

**Gene Set Enrichment Analysis (GSEA)**

GSEA was carried out to determine which pathways and biological processes were enriched among proteins differentially abundant between baseline CHE patients over healthy donor controls, and other groups of samples. Standard ranking by log_2_ fold change alone may cause tied values for proteins with identical expression changes, and ranking solely by adjusted *P*-value may be biased when sample sizes differ between groups. To address these issues, we developed a Composite Score (CS) that combines both the magnitude of change and the statistical confidence for each protein (R. EKREN, Companion to “Redefining chronic hand eczema as a type 2 disease: results from a phase 2b randomized controlled trial comparing dupilumab and placebo” study. (2024) (available at https://zenodo.org/records/13918363)).

***Notation***

*R_i_: log2 fold change for protein I	p_adj_i_: Adjusted P value for protein i*

*R_min_, R_max_: Minimum and maximum observed log2 fold changes for the given protein*

- **Step 4.1: Normalization of Fold Changes** To standardize effect sizes to a [0, 1] interval, log2 fold changes (*R*_i_) were processed using min–max scaling. In the specific instance where the range (*R*_max_ – *R*_min_) equaled zero, the normalized value (*NormFC*_i_) was set to 0. Otherwise, it was calculated as: *NormFC*_i_ = (*R*_i_ – *R*_min_) / (*R*_max_ – *R*_min_)
- **Step 4.2: Transformation of Significance Statistics** To prioritize robust statistical detections, adjusted P values were log-transformed. A pseudocount of 10^–10^ was included to prevent undefined values when p_adj_i_ was zero: *T***_i_ =** –log_10_(p_adj_i_ + 10^–10^)
- **Step 4.3: Determination of Weighting Factor** A weighting factor λ was calculated to balance the contribution of statistical significance relative to the distribution of fold changes. This factor was defined as the reciprocal of the fold change range (1 / (R_max_ – R_min_)), or set to 1 if the range was zero.
- **Step 4.4: Final Score Calculation** The final Composite Score (CS_i_) was computed by integrating the normalized magnitude and the weighted statistical significance using the following multiplicative model: CS_i_ = *NormFC*_i_ × [1 + (λ × T_i_)]
- **Step 4.5: Enrichment Testing** Proteins ranked by CS were analyzed using clusterProfiler R package (S. Xu, E. Hu, Y. Cai, Z. Xie, X. Luo, L. Zhan, W. Tang, Q. Wang, B. Liu, R. Wang, W. Xie, T. Wu, L. Xie, G. Yu, Using clusterProfiler to characterize multiomics data. *Nat. Protoc.* (2024), doi:10.1038/s41596-024-01020-z), employing the MSigDB "C5: GO:BP" collection. Protein identifiers were mapped to official gene symbols and significant enrichment was determined with Benjamini-Hochberg adjusted P-value <0.05 (gene sets size 15–500). Bubble plots displaying Normalized Enrichment Score (NES) (color) and -log_10_*P*_adj_ (size). Pathway names were formatted to enhance readability.

**5. Skin total RNA sequencing**

**RNA extraction**

Samples were collected into RNAlaterTM (Qiagen) and stored at 4°C for 48 hours after collection and transferred to -80 °C until RNA extraction. For RNA extraction, each skin sample was transferred in a gentleMACS M Tube (Miltenyi) filled with 700 μl of TRIzol (Invitrogen) and put on ice. Sample was then dissociated using the GentleMAX^TM^ Octo Dissociator (Miltenyi, Program: RNA_1) and centrifuged shortly. The content of the M tube was then transferred to a 1.5 mL tube and 140 μl of chloroform was added. Tube was vortexed for 15 seconds twice and then centrifuged for 15 minutes at 8000 rpm at 4°C. The upper phase of the tube was collected and transferred to a new 1.5 mL tube filled with 300 μL of 70% ethanol, homogenized and put on ice. The homogenized lysate was transferred on rNeasy spin column placed in a 2 ml collection tube and processed using the RNAeasy Plus Mini KIT (Qiagen #74104) according to the manufacturer instructions. The RNA collected were stored at -80°C until sequencing.

**RNA Quality Control**

Samples quality controls and libraries preparation were performed at the GeT-Santé facility (Inserm, Toulouse, France, get.genotoul.fr). RNA concentration and purity were determined using a ND-2000 149 Spectrophotometer (Thermo Fisher Scientific, Waltham, USA). Integrity of RNA was checked with a Fragment Analyzer (Agilent Technologies, Santa Clara, USA), using the RNA Standard Sensitivity Kit. The cut-off for RIN was 9 and all the samples in a similar range.

**Library preparation and quantification**

RNA-seq paired-end libraries were prepared according to Illumina’s protocol with some adjustments, using the TruSeq Stranded mRNA library prep Kit (Illumina, San Diego, USA). Briefly, mRNAs were first selected from 1000 ng total RNA using poly-T beads. Then, RNA was fragmented during 2 sec and retrotranscribed to generate double stranded cDNA. Compatible adaptors were ligated, allowing the barcoding of the samples with unique dual indices. 12 cycles of PCR were applied to amplify libraries, and a final purification step allowed to obtain 280-1000 pb fragments. Libraries quality was assessed using the HS NGS kit on the Fragment Analyzer (Agilent Technologies, Santa Clara, USA). Libraries quantification and sequencing were performed at the GeT-PlaGe core facility (INRAE, Toulouse, France). Libraries were quantified by qPCR using the KAPA Library Quantification Kit (Roche, Basel, Switzerland) to obtain an accurate quantification.

**Sequencing**

Libraries were equimolarly pooled and RNA sequencing was then performed on one S4 lane of the Illumina NovaSeq 6000 instrument (Illumina, San Diego, USA), using the NovaSeq 6000 S4 v1.5 Reagent Kit (300 cycles), and a paired-end 2 x 150 pb strategy.

**Alignment and RNA-Seq Quantification.**

Fastq files processed using nf-core/rnaseq (v3.18.0) of the nf-core collection of workflows (P. A. Ewels, A. Peltzer, S. Fillinger, H. Patel, J. Alneberg, A. Wilm, M. U. Garcia, P. Di Tommaso, S. Nahnsen, The nf-core framework for community-curated bioinformatics pipelines. *Nat. Biotechnol.* **38**, 276–278 (2020)), utilizing reproducible software environments from the Bioconda (B. Grüning, R. Dale, A. Sjödin, B. A. Chapman, J. Rowe, C. H. Tomkins-Tinch, R. Valieris, J. Köster, Bioconda Team, Bioconda: sustainable and comprehensive software distribution for the life sciences. *Nat. Methods* **15**, 475–476 (2018)) and Biocontainers (F. da Veiga Leprevost, B. A. Grüning, S. Alves Aflitos, H. L. Röst, J. Uszkoreit, H. Barsnes, M. Vaudel, P. Moreno, L. Gatto, J. Weber, M. Bai, R. C. Jimenez, T. Sachsenberg, J. Pfeuffer, R. Vera Alvarez, J. Griss, A. I. Nesvizhskii, Y. Perez-Riverol, BioContainers: an open-source and community-driven framework for software standardization. *Bioinforma. Oxf. Engl.* **33**, 2580–2582 (2017)) projects. The alignment and quantification of the reads were executed within Nextflow v23.10.0 (P. Di Tommaso, M. Chatzou, E. W. Floden, P. P. Barja, E. Palumbo, C. Notredame, Nextflow enables reproducible computational workflows. *Nat. Biotechnol.* **35**, 316–319 (2017)) with the following command:

nextflow run nf-core/rnaseq -r 3.18.0 -profile genotoul --input samplesheet.csv --outdir alignNquant/ ---fasta /home/rekren/save/refs/Homo_sapiens.GRCh38.111.dna_rm.toplevel.fa.gz --gtf /home/rekren/save/refs/Homo_sapiens.GRCh38.111.gtf.gz --remove_ribo_rna --aligner star_salmon --star_index /home/rekren/save/refs/genome/index/star/ --salmon_index /home/rekren/save/refs/genome/index/salmon/ --extra_star_align_args '--alignIntronMax 1000000 --alignIntronMin 20 --alignMatesGapMax 1000000 --alignSJoverhangMin 8 --outFilterMismatchNmax 10 --outFilterType BySJout --outFilterMismatchNoverLmax 0.05' --multiqc_title DupEczeMain Alignment and QC assesment

Thus, pre-processing of the reads was completed also by including the removing PCR duplicates, removing poor quality reads, removal of ribosomal RNAs and determination of quantified gene expressions of the samples. Quantified gene counts and transcript lengths were imported from the nf-core/rnaseq (v3.18.0) pipeline’s output.

**Bulk RNA-seq Data Analysis Algorithm**

Downstream differential expression analysis was conducted using DESeq2 R package (M. I. Love, W. Huber, S. Anders, Moderated estimation of fold change and dispersion for RNA-seq data with DESeq2. *Genome Biol.* **15**, 550 (2014)) according to the following algorithmic steps :

- **Step 5.1: Statistical Modeling and Normalization.** A DESeqDataSet was created integrating transcript lengths as an additional normalization factor. Pairwise contrasts were generated based on the experimental setup (i.e., baseline CHE patients versus Healthy donor controls). The modeling was run utilizing the Wald test with a parametric fit, enabling parallel processing on four cores. Outlier replacement was performed with a minimum threshold of seven replicates, and size factors were estimated using the "ratio" method.
- **Step 5.2: Variance Stabilization and Shrinkage**. Variance Stabilizing Transformation (VST) was applied to stabilize variance across the data. Subsequently, log2 fold changes (LFC) were shrunken using the adaptive shrinkage (ashr) method selection.
- **Step 5.3: Dimensionality Reduction.** Principal Component Analysis (PCA) was performed on the top 1000 hypervariable genes. The first 2 Principal Components (PCs) were plotted with samples colored by condition. Ellipses were drawn as polygons representing a 0.99 confidence level, with transparency and size adjusted for optimal visibility of data points. The PCA plot provides a visual representation of the differences in gene expression patterns between the groups.
- **Step 5.4: Visualization of Differentially Expressed Genes (DEGs).** Significance was defined as Adjusted *P* < 0.05 and |shrunken log2 fold change| ≥ 1. Shrunken LFC versus significance was visualized in Volcano plots using the ggplot2 R package (H. Wickham, ggplot2 (2016) (available at http://link.springer.com/10.1007/978-3-319-24277-4)). For heatmaps, the top 50 DEGs were retrieved based on significance thresholds, and VST-normalized counts were z-score standardized. Hierarchical clustering was performed using Euclidean distance and the "ward.D2" method implemented in the stats package (M. Dia, T. C. Wehner, C. Arellano, RGxE: An R Program for Genotype x Environment Interaction Analysis. *Am. J. Plant Sci.* **08**, 1672–1698 (2017)). This approach allows for the visualization and identification of distinct sample clusters based on their gene expression profiles.
- **Step 5.5: Gene Set Enrichment Analysis** Differential expression results were ranked based on log2 fold changes. Gene sets were retrieved MSigDB "C5: GO:BP" collection. "C5, GO:BP" and analyzed via clusterProfiler R package (X. Zhang, A. H. Smits, G. B. van Tilburg, H. Ovaa, W. Huber, M. Vermeulen, Proteome-wide identification of ubiquitin interactions using UbIA-MS. *Nat. Protoc.* **13**, 530–550 (2018)). Terms from mSigDB were mapped to Ensembl gene identifiers, and adjusted *P* values were calculated using the Bonferroni correction method adjusted P-value <0.05 (gene sets size 15–500). Bubble plots displaying Normalized Enrichment Score (NES) (color) and -log_10_*P*_adj_ (size). Pathway names were formatted to enhance readability.
- **Step 5.6: Comparative Analysis and Overlap Calculation.** The percentage of overlap between DEGs identified in our CHE cohort at baseline and DEGs extracted from publicly available datasets (AD, CP, PPPso, ACD, ICD) was calculated by using the formula: (Shared DEGs / Total Reference CHE DEGs) × 100. Analysis was restricted to canonical biomarkers of type 1, 2, and 3 immune polarization based on recent publications in the context of inflammatory, atopic, allergic, and irritant skin diseases (31–38). Only DEGs that were consistently and directionally (up- or downregulated) reported in at least two published datasets of patients with AD, psoriasis (CP or PPPso), ACD, or ICD (31–36) were retained for comparative analyses.

**6. Clinical Statistical Analysis**

The statistical analysis plan was run using STATA software (Release 18.0, Stata Corporation), with significance defined as a two-sided P value < 0.05. The workflow proceeded through five distinct analytical phases:

- **Step 6.1: Sample Size Determination and Power Calculation.** The required sample size was calculated to detect a clinically significant absolute difference of -25% in the mean percentage change of the mTLSS score at week 16. Assuming a standard deviation of 40% in both arms, a total of 84 patients (42 per group) was determined to provide >80% power with a type-I error of 0.05 using a two-sided two-sample t-test (J. F. Fowler, O. Graff, A. G. Hamedani, A phase 3, randomized, double-blind, placebo-controlled study evaluating the efficacy and safety of alitretinoin (BAL4079) in the treatment of severe chronic hand eczema refractory to potent topical corticosteroid therapy. *J. Drugs Dermatol. JDD* **13**, 1198–1204 (2014)). To account for an estimated 10% of patients who might not be evaluable, it was necessary to randomize a total of 94 patients.
- **Step 6.2: Baseline Characterization.** Baseline characteristics, including disease assessment, were analyzed using descriptive statistics (e.g., mean and standard deviation (SD) for continuous variables, or number and frequency tabulations for categorical variables) for each group. Baseline homogeneity between groups of these variables was assessed descriptively (M. R. de Boer, W. E. Waterlander, L. D. J. Kuijper, I. H. M. Steenhuis, J. W. R. Twisk, Testing for baseline differences in randomized controlled trials: an unhealthy research behavior that is hard to eradicate. *Int. J. Behav. Nutr. Phys. Act.* **12**, 4 (2015)).
- **Step 6.3: Efficacy Analysis Modeling.** The primary efficacy analysis (consisted of a comparison of the mean percentage change in mTLSS at week 16 was performed on the the Intention-to-Treat (ITT) population. A multiple linear regression model was constructed to control for randomization stratification factor (history of atopic dermatitis) and baseline score. Interaction between treatment arm and stratification variable was tested and found no significant, indicating that the treatment effect was not significantly different according to history of atopic dermatitis. Missing data on mTLSS score at week 16 were handled using the Last-Observation-Carry-Forward (LOCF) imputation method. The same approach was used to assess changes from baseline to week 16 in EASI, DLQI, EQ-VAS, WPAI, and VAS for pruritus, sleep loss, and pain. We also conducted a Cochran-Mantel-Haenszel test to evaluate whether dupilumab (compared to placebo) had an effect on the IGA and PaGA scores of 0/1 (meaning cleared/almost cleared CHE), while controlling for the stratification factor used in randomization. Sensitivity analyses were conducted for the comparison of the mean percentage change in mTLSS at week 16 using Multiple Imputation method and Complete-Case Analysis.
- **Step 6.4: Clinical Dimensionality Reduction and Clustering.** To visualize patient heterogeneity based on clinical features, PCA was performed using the “stats” R package. Following dimensionality reduction, hierarchical clustering was performed utilizing Euclidean distances (calculated via the “dist” function) and the "ward.D2" agglomeration method (“hclust” function). Clusters were visualized on the first two principal components, with treatment-based coloring and 95% confidence ellipses.
- **Step 6.5: Safety Analysis**. Safety analyses were conducted on the treated population. Adverse events were classified according to the MedDRA System Organ Class (SOC) and Preferred Term (PT). The incidence (number and percentage) of patients reporting adverse events was presented by group, accompanied by exact 95% Confidence Intervals (CI) calculated from study start to study end.

**Data and materials availability**

All the processed data of gene expression, proteins and codes for the analysis is wrapped up in one repository (R. EKREN, Companion to “Redefining chronic hand eczema as a type 2 disease: results from a phase 2b randomized controlled trial comparing dupilumab and placebo” study. (2024) (available at https://zenodo.org/records/13918363)). Companion to "Integrated Clinical Trial and Molecular Profiling Reveals Immune Drivers of Chronic Hand Eczema” study. Zenodo. <https://doi.org/10.5281/zenodo.13918363>. The raw and processed datasets are deposited under the GSE279422, and will be made available upon acceptance of the manuscript.

|  | | **Placebo arm** | **Dupilumab arm** | **Total** | |
| --- | --- | --- | --- | --- | --- |
|  |  | N=47 (50%) | N=47 (50%) | N=94 (100%) | |
| **Demographic characteristics** | | | | |  |
| **Gender, n (%)** | | | | | |
| Male | | 23 (48.9%) | 14 (29.8%) | 37 (39.4%) | |
| Female | | 24 (51.1%) | 33 (70.2%) | 57 (60.6%) | |
| **Age in years (mean (SD))** | | 41.1 (15.86) | 37.8 (13.60) | 39.5 (14.79) | |
| **Phototype, n (%)** | |  |  |  | |
| 1-4 | | 45 (95.8%) | 46 (97.9%) | 91 (96.8%) | |
| 5-6 | | 2 (4.2%) | 1 (2.1%) | 3 (3.2%) | |
| **Profession, n (%)** | | | | | |
| Office worker | | 18 (38.3%) | 19 (40.4%) | 37 (39.4%) | |
| Healthcare professional | | 5 (10.6%) | 6 (12.8%) | 11 (11.7%) | |
| Manual workers (construction, mechanics, electrician, wood) | | 5 (10.6%) | 4 (8.5%) | 9 (9.6%) | |
| Restauration | | 4 (10.6%) | 4 (8.5%) | 9 (9.6%) | |
| Cleaning agent | | 3 (6.4%) | 5 (10.6%) | 8 (8.5%) | |
| Hairdresser, Beautician | | 1 (2.1%) | 1 (2.1%) | 2 (2.1%) | |
| Farmer | | 1 (2.1%) | 1 (2.1%) | 2 (2.1%) | |
| Retired/Unemployed | | 6 (12.8%) | 6 (12.8%) | 12 (12.8%) | |
| Other | | 3 (6.4%) | 1 (2.1%) | 4 (4.3%) | |
| **History of personal atopy, n (%)** | | 37 (78.7%) | 41 (87.2%) | 78 (83.0%) | |
| Atopic dermatitis | | 30 (63.8%) | 34 (72.3%) | 64 (68.1%) | |
| Allergic rhinitis | | 28 (59.6%) | 25 (53.2%) | 53 (56.4%) | |
| Asthma | | 17 (36.2%) | 16 (34.0%) | 33 (35.1%) | |
| Allergic conjunctivitis | | 14 (29.8%) | 12 (25.5%) | 26 (27.7%) | |
| Food allergy | | 5 (10.6%) | 10 (21.3%) | 15 (16.0%) | |
| Known sensitization to pneumallergens (prick or IgE-confirmed) | | 23 (48.9%) | 20 (42.6%) | 43 (45.7%) | |
| **Family history of atopy, n (%)** | | 22 (46.8%) | 30 (63.8%) | 52 (55.3%) | |
| **History of CHE disease** | | | | |  |
| **Time since diagnosis in years (mean (SD))** | | 9.24 (13.037) | 8.20 (8.909) | 8.72 (11.118) | |
| **Previous treatment for CHE** | Therapeutic education, n (%) | 9 (19.1%) | 8 (17.0%) | 17 (18.1%) | |
|  | Potent or very potent topical corticosteroids, n (%) | 47 (100.0%) | 47 (100.0%) | 94 (100.0%) | |
|  | Topical tacrolimus, n (%) | 13 (28.9%) | 18 (40.0%) | 31 (34.4%) | |
|  | Ciclosporin, n (%) | 3 (6.4%) | 2 (4.3%) | 5 (5.3%) | |
|  | *Duration in months (mean (SD))* | 10.67 (4.726) | 19.5 (6.364) | 14.2 (6.686) | |
|  | Methotrexate, n (%) | 6 (12.8%) | 1 (2.1%) | 7 (7.4%) | |
|  | *Duration in months (mean (SD))* | 24.5 (46.805) | 12.00 (0.0) | 22.71 (42.987) | |
|  | Alitretinoin, n (%) | 12 (25.5%) | 14 (29.8%) | 26 (27.7%) | |
|  | *Duration in months (mean (SD))* | 5.83 (7.017) | 6.93 (6.306) | 6.42 (6.531) | |
|  | Oral corticosteroid, n (%) | 5 (10.6%) | 8 (17.0%) | 13 (13.8%) | |
|  | *Duration in months (mean (SD))* | 0.9 (0.224) | 0.71 (0.756) | 0.79 (0.582) | |
|  | Phototherapy, n (%) | 5 (11.1%) | 6 (13.3%) | 11 (12.2%) | |
|  | *Nb of sessions (mean (SD))* | 23.33 (11.547) | 34.80 (17.541) | 30.50 (15.784) | |
| **Irritative aggravating cofactors, n (%)** | | 22 (46.8%) | 22 (46.8%) | 44 (46.8%) | |
| **Allergic aggravating cofactors, n (%)** | | 6 (12.8%) | 5 (10.6%) | 11 (11.7%) | |
| **Working test** *(active CHE at work and improvement/ regression offsite work),* **n (%)** | |  |  |  | |
| Positive | | 2 (5.9%) | 5 (12.5%) | 7 (9.5%) | |
| Negative | | 25 (73.5%) | 27 (67.5%) | 52 (70.3%) | |
| Doubtful | | 7 (20.6%) | 8 (20.0%) | 15 (20.3%) | |
| **Currently off work due to CHE, n (%)** | | 5 (10.6%) | 7 (14.9%) | 12 (12.8%) | |

**Supplementary Table 1. Baseline patients characteristics.** CHE: chronic hand eczema, SD: standard deviation.

|  | | **Placebo arm** | **Dupilumab arm** | **Total** |  |
| --- | --- | --- | --- | --- | --- |
|  |  | N=47 (50%) | N=47 (50%) | N=94 (100%) |  |
| **CHE examination** | | | | | |
| Unilateral involvement, n (%) | | 1 (2.1%) | 3 (6.4%) | 4 (4.3%) |  |
| Bilateral involvement, n (%) | | 46 (97.9%) | 44 (93.6%) | 90 (95.7%) |  |
| Palmar involvement only, n (%) | | 6 (12.8%) | 6 (12.8%) | 12 (12.8%) |  |
| Dorsal involvement only, n (%) | | 5 (10.6%) | 5 (10.6%) | 10 (10.6%) |  |
| Palmar and dorsal involvements, n (%) | | 36 (76.6%) | 36 (76.6%) | 72 (76.6%) |  |
| Vesicles, n (%) | | 20 (42.6%) | 18 (38.3%) | 38 (40.4%) |  |
| Cracks, n (%) | | 34 (72.3%) | 30 (63.8%) | 64 (68.1%) |  |
| Hyperkeratosis, n (%) | | 31 (66.0%) | 29 (61.7%) | 60 (63.8%) |  |
| Pulpitis, n (%) | | 22 (46.8%) | 21 (44.7%) | 43 (45.7%) |  |
| Nails affected, n (%) | | 13 (27.7%) | 20 (42.6%) | 33 (35.1%) |  |
| Eczema on feet*, n (%) | | 18 (38.3%) | 10 (21.3%) | 28 (29.8%) |  |
| **Disease severity scoring** | | | | | |
| mTLSS - Modified total lesion symptom score (mean (SD)) / 25 | | 12.5 (2.71) | 12.0 (3.15) | 12.2 (2.93) |  |
| IGA - Investigator global assessment (mean (SD)) / 4 | | 3.3 (0.48) | 3.3 (0.51) | 3.3 (0.49) |  |
| PaGA - Patient Global Assessment of disease severity (mean (SD)) / 4 | | 3.3 (0.67) | 3.3 (0.66) | 3.3 (0.66) |  |
| Visual analogue scales of pruritus (mean (SD)) / 100 | | 63.1 (23.63) | 60.8 (29.73) | 62.0 (26.70) |  |
| Visual analogue scales of pain (mean (SD)) / 100 | | 53.5 (27.61) | 60.2 (27.32) | 56.8 (27.53) |  |
| Visual analogue scales of sleep loss (mean (SD)) / 100 | | 41.3 (27.37) | 39.8 (30.52) | 40.5 (28.85) |  |
| EASI – Eczema area and severity index (mean (SD)) / 72 | | 7.13 (5.969) | 5.36 (4.100) | 6.3 (5.216) |  |
| EQ-VAS – EuroQol-visual analogue scales (mean (SD)) / 100 | | 61.8 (19.43) | 68.9 (21.20) | 65.3 (20.54) |  |
| DLQI - Dermatology life quality index (mean (SD)) / 30 | | 12.1 (5.38) | 13.0 (5.21) | 12.5 (5.28) |  |
| WPAI - Work productivity and activity impairment questionnaire | Work time missed (%) (mean (SD)) | 10.1 (28.83) | 8.9 (26.16) | 9.4 (27.10) |  |
|  | Impairment while working due to health (%) (mean (SD)) | 33.5 (25.34) | 41.6 (25.31) | 38.1 (25.41) |  |
|  | Overall work impairment due to health (%) (mean (SD)) | 40.8 (29.45) | 46.7 (27.86) | 44.1 (28.45) |  |
|  | Activity impairment due to health (%) (mean (SD)) | 50.9 (25.89) | 57.8 (25.31) | 54.3 (25.70) |  |
| **Biological routine atopic traits** | | | | | |
| **Eosinophils blood count (G/L)** | |  |  |  |  |
| Mean (SD) | | 0.24 (0.118) | 0.17 (0.139) | 0.20 (0.132) |  |
| Min ; Max | | 0.1 ; 0.5 | 0.0 ; 0.8 | 0.0 ; 0.8 |  |
| **Total IgE serum level (kUI/L)** | |  |  |  |  |
| Mean (SD) | | 858.34 (1493.248) | 236.38  (365.017) | 564.83 (1151.150) |  |
| > 150 | | 21 (44.7%) | 26 (61.9%) | 47 (52.8%) |  |
| < 150 | | 26 (55.3%) | 16 (38.1%) | 42 (47.2%) |  |

**Supplementary Table 2. Baseline CHE disease characteristics.** *Plantar or dorsal involvement not detailed. IgE: Immunoglobulin E, CHE: chronic hand eczema, SD: standard deviation, VAS: Visual analogue scales.

|  | | | **< 63.64% improvement** | | **≥ 63.64% improvement** | **Total** | | **p-value** |  |
| --- | --- | --- | --- | --- | --- | --- | --- | --- | --- |
|  |  |  | (N=23) | | (N=24) | (N=47) | |  |  |
| **Demographic characteristics** | | | | | | | | |  |
| **Gender, n (%)** | | | | | | | | |  |
| Male | | | 6 (26.1%) | | 8 (33.3%) | 14 (29.8%) | | 0.587 |  |
| Female | | | 17 (73.9%) | | 16 (66.7%) | 33 (70.2%) | |  |  |
| **Age in years (mean (SD))** | | | 34.13 (11.514) | | 41.38 (14.708) | 37.83 (13.600) | | 0.067 |  |
| **Phototype, n (%)** | | | | | | | | |  |
| 1-4 | | | 22 (95.7%) | | 24 (100.0%) | 46 (97.9%) | | 0.489 |  |
| 5-6 | | | 1 (4.3%) | | - | 1 (2.1%) | |  |  |
| **History of personal atopy, n (%)** | | | 19 (82.6%) | | 22 (91.7%) | 41 (87.2%) | | 0.416 |  |
| Atopic dermatitis | | | 16 (69.6%) | | 18 (75.0%) | 34 (72.3%) | | 0.67 |  |
| Allergic rhinitis | | | 10 (43.5%) | | 15 (62.5%) | 25 (53.2%) | | 0.191 |  |
| Asthma | | | 7 (30.4%) | | 9 (37.5%) | 16 (34.0%) | | 0.609 |  |
| Allergic conjunctivitis | | | 6 (26.1%) | | 6 (25.0%) | 12 (25.5%) | | 0.932 |  |
| Food allergy | | | 4 (17.4%) | | 6 (25.0%) | 10 (21.3%) | | 0.724 |  |
| Known sensitization to pneumallergens (prick or IgE-confirmed) | | | 8 (34.8%) | | 12 (50.0%) | 20 (42.6%) | | 0.292 |  |
| **Family history of atopy, n (%)** | | | 14 (60.9%) | | 16 (66.7%) | 30 (63.8%) | | 0.679 |  |
| **History of CHE disease** | | | | | | | | |  |
| **Time since diagnosis in years (mean (SD))** | | | 9.98 (7.713) | | 6.48 (9.777) | 8.20 (8.909) | | 0.181 |  |
| **Previous treatment for CHE, n (%)** | Therapeutic education | | 2 (8.7%) | | 6 (25.0%) | 8 (17.0%) | | 0.245 |  |
|  | Topical tacrolimus | | 6 (28.6%) | | 12 (50.0%) | 18 (40.0%) | | 0.143 |  |
|  | Ciclosporin | | 1 (4.3%) | | 1 (4.2%) | 2 (4.3%) | | 1.000 |  |
|  | Methotrexate | | 1 (4.3%) | | 0 (0.0%) | 1 (2.1%) | | 0.489 |  |
|  | Alitretinoin | | 7 (30.4%) | | 7 (29.2%) | 14 (29.8%) | | 0.924 |  |
|  | Oral corticosteroid | | 3 (13.0%) | | 5 (20.8%) | 8 (17.0%) | | 0.701 |  |
|  | Phototherapy | | 2 (9.5%) | | 4 (16.7%) | 6 (13.3%) | | 0.670 |  |
| **Irritative aggravating cofactors, n (%)** | | | 9 (39.1%) | | 13 (54.2%) | 22 (46.8%) | | 0.302 |  |
| **Allergic aggravating cofactors, n (%)** | | | 1 (4.3%) | | 4 (16.7%) | 5 (10.6%) | | 0.348 |  |
| **Working test** *(active CHE at work and improvement/ regression offsite work),* **n (%)** | | | | | | | | |  |
| Positive | | | 2 (10.0%) | | 3 (15.0%) | 5 (12.5%) | | 1.000 |  |
| Negative | | | 14 (70.0%) | | 13 (65.0%) | 27 (67.5%) | |  |  |
| Doubtful | | | 4 (20.0%) | | 4 (20.0%) | 8 (20.0%) | |  |  |
| **Currently off work due to CHE, n (%)** | | | 2 (8.7%) | | 5 (20.8%) | 7 (14.9%) | | 0.416 |  |
| **CHE examination** | | | | | | | | | |
| Unilateral involvement, n (%) | | | | 3 (13.0%) | 0 (0.0%) | 3 (6.4%) | 0.109 | | |
| Bilateral involvement, n (%) | | | | 20 (87.0%) | 24 (100.0%) | 44 (93.6%) |  |  |  |
| Palmar involvement only, n (%) | | | | 1 (4.3%) | 5 (20.8%) | 6 (12.8%) | 0.016 | | |
| Dorsal involvement only, n (%) | | | | 5 (21.7%) | 0 (0.0%) | 5 (10.6%) |  |  |  |
| Palmar and dorsal involvements, n (%) | | | | 17 (73.9%) | 19 (79.2%) | 36 (76.6%) |  |  |  |
| Vesicles, n (%) | | | | 7 (30.4%) | 11 (45.8%) | 18 (38.3%) | 0.278 | | |
| Cracks, n (%) | | | | 16 (69.6%) | 14 (58.3%) | 30 (63.8%) | 0.423 | | |
| Hyperkeratosis, n (%) | | | | 15 (65.2%) | 14 (58.3%) | 29 (61.7%) | 0.627 | | |
| Pulpitis, n (%) | | | | 9 (39.1%) | 12 (50.0%) | 21 (44.7%) | 0.454 | | |
| Nails affected, n (%) | | | | 10 (43.5%) | 10 (41.7%) | 20 (42.6%) | 0.900 | | |
| Eczema on feet, n (%) | | | | 5 (21.7%) | 5 (20.8%) | 10 (21.3%) | 1.000 | | |
| **Disease severity scoring** | | | | | | | | | |
| mTLSS - Modified total lesion symptom score (mean (SD)) / 25 | | | | 11.6 (3.54) | 12.4 (2.75) | 12.0 (3.15) | 0.410 | | |
| IGA - Investigator global assessment (mean (SD)) / 4 | | | | 3.3 (0.56) | 3.3 (0.46) | 3.3 (0.51) | 0.933 | | |
| PaGA - Patient Global Assessment of disease severity (mean (SD)) / 4 | | | | 3.3 (0.63) | 3.3 (0.70) | 3.3 (0.66) | 0.760 | | |
| Visual analogue scales of pruritus (mean (SD)) / 100 | | | | 62.0 (28.29) | 59.7 (31.65) | 60.8 (29.73) | 0.798 | | |
| Visual analogue scales of pain (mean (SD)) / 100 | | | | 61.6 (26.62) | 58.8 (28.45) | 60.2 (27.32) | 0.732 | | |
| Visual analogue scales of sleep loss (mean (SD)) / 100 | | | | 37.4 (30.20) | 42.1 (31.28) | 39.8 (30.52) | 0.603 | | |
| EASI – Eczema area and severity index (mean (SD)) / 72 | | | | 4.43 (3.309) | 6.227 (4.667) | 5.36 (4.100) | 0.266 | | |
| EQ-VAS – EuroQol-visual analogue scales (mean (SD)) / 100 | | | | 77.4 (11.48) | 61.2 (25.07) | 68.9 (21.20) | 0.019 | | |
| DLQI - Dermatology life quality index (mean (SD)) / 30 | | | | 13.1 (5.17) | 12.8 (5.35) | 13.0 (5.21) | 0.869 | | |
| WPAI - Work productivity and activity impairment questionnaire | | Work time missed (%) (mean (SD)) | | 9.4 (27.20) | 8.5 (25.98) | 8.9 (26.16) | 0.929 | | |
|  |  | Impairment while working due to health (%) (mean (SD)) | | 34.7 (23.26) | 48.1 (26.13) | 41.6 (25.31) | 0.142 | | |
|  |  | Overall work impairment due to health (%) (mean (SD)) | | 41.6 (26.94) | 51.4 (28.66) | 46.7 (27.86) | 0.316 | | |
|  |  | Activity impairment due to health (%) (mean (SD)) | | 56.4 (24.98) | 59.1 (26.10) | 57.8 (25.31) | 0.718 | | |
| **Biological routine atopic traits** | | | | | | | | | |
| **Eosinophils blood count (G/L)** | | | |  |  |  |  | | |
| Mean (SD) | | | | 0.19 (0.111) | 0.16 (0.162) | 0.17 (0.139) | 0.421 | | |
| Min ; Max | | | | 0.0 ; 0.4 | 0.0 ; 0.8 | 0.0 ; 0.8 |  |  |  |
| **Total IgE serum level (kUI/L)** | | | |  |  |  |  | | |
| Mean (SD) | | | | 324.90 (469.767) | 147.86 (189.735) | 236.38 (365.017) | 0.624 | | |
| > 150 | | | | 12 (57.1%) | 14 (66.7%) | 26 (61.9%) | 0.525 | | |
| < 150 | | | | 9 (42.9%) | 7 (33.3%) | 16 (38.1%) |  |  |  |

**Supplementary Table 3. Analysis of factors influencing dupilumab efficacy based on primary endpoint response.**

This table presents the analysis of factors influencing dupilumab efficacy in patients with chronic hand eczema (CHE), comparing those who achieved greater than 63.64% improvement (above the median) with those who achieved less than 63.64% improvement (below the median). The median improvement of 63.64% was used as a threshold to categorize patients based on their response to treatment.

| Patients included in the molecular study | Group | Analysis performed | | | | | | | | History of AD | Irritative factors | Proven contact allergy | mTLSS | | IGA | |
| --- | --- | --- | --- | --- | --- | --- | --- | --- | --- | --- | --- | --- | --- | --- | --- | --- |
|  |  | Skin total RNA sequencing | | | Blood immune cells phenotyping by spectral cytomety | Skin LC-MS/MS shotgun proteomic | | Serum LC-MS/MS shotgun proteomic | NULISAseq - Alamar |  |  |  | W0 | W16 | W0 | W16 |
|  |  | x | Matched healthy donor skin | Biopsy site |  | x | Matched healthy donor skin |  |  |  |  |  |  |  |  |  |
| P1 | Placebo | x | From abdomen or arm  n = 15, and from hand palm n = 10 | P/D interface |  |  | From the back of the hand  n=4 |  | x | yes | no | no | 11 | 8 | 3 | 2 |
| P2 |  | x |  | D | x |  |  |  | x | no | yes | no | 15 | 8 | 4 | 3 |
| P3 |  | x |  | D | x |  |  |  | x | yes | no | no | 15 | 12 | 4 | 3 |
| P4 |  | x |  | D | x |  |  |  | x | yes | no | no | 13 | 11 | 3 | 3 |
| P6 |  | x |  | P | x |  |  |  | x | no | yes | yes | 15 | 13 | 3 | 3 |
| P9 |  | x |  | D | x |  |  |  |  | yes | no | yes | 10 |  | 3 |  |
| P13 |  |  |  |  | x |  |  |  | x | yes | no | no | 14 | 17 | 4 | 4 |
| P14 |  |  |  |  | x |  |  |  | x | no | yes | no | 17 | 12 | 4 | 4 |
| P15 |  |  |  |  | x |  |  |  | x | yes | no | no | 9 | 6 | 3 | 2 |
| P17 |  |  |  |  | x |  |  |  | x | yes | yes | yes | 14 | 11 | 3 | 3 |
| P18 |  |  |  |  | x |  |  |  | x | yes | yes | yes | 14 | 2 | 3 | 1 |
| P20 |  |  |  |  |  |  |  |  | x | yes | yes | no | 18 | 15 | 4 | 4 |
| P21 |  |  |  |  | x |  |  | x |  | yes | yes | no | 11 | 19 | 3 | 4 |
| P41 |  |  |  | P/D interface |  | x |  | x | x | no | yes | yes | 12 | 13 | 3 | 3 |
| P42 |  |  |  | P/D interface | x | x |  | x | x | yes | no | no | 12 |  | 3 |  |
| P43 |  |  |  | D |  | x |  | x | x | yes | no | no | 18 | 15 | 4 | 4 |
| P47 |  | x |  | D | x |  |  |  | x | yes | no | yes | 14 |  | 3 |  |
| D1 | Dupilumab | x |  | P | x |  |  |  | x | yes | yes | yes | 17 | 5 | 4 | 1 |
| D2 |  | x |  | P/D interface |  |  |  |  | x | yes | yes | yes | 9 | 3 | 3 | 1 |
| D3 |  |  |  |  |  |  |  |  | x | no | no | no | 14 | 3 | 4 | 1 |
| D4 |  | x |  | D | x |  |  |  |  | yes | yes | no | 10 | 5 | 3 | 2 |
| D5 |  | x |  | D |  |  |  |  | x | yes | yes | no | 12 | 7 | 4 | 2 |
| D6 |  | x |  | D | x |  |  |  | x | no | no | yes | 14 | 6 | 4 | 2 |
| D7 |  |  |  |  | x |  |  |  | x | yes | yes | no | 10 | 3 | 3 | 1 |
| D8 |  | x |  | D | x |  |  |  | x | yes | no | yes | 13 | 2 | 3 | 1 |
| D10 |  | x |  | P/D interface | x |  |  |  | x | yes | yes | no | 12 | 2 | 3 | 1 |
| D12 |  | x |  | P |  |  |  |  | x | no | no | yes | 7 | 4 | 3 | 2 |
| D14 |  |  |  |  | x |  |  |  | x | no | no | yes | 15 | 1 | 4 | 0 |
| D15 |  |  |  |  | x |  |  |  | x | yes | no | no | 16 | 6 | 4 | 1 |
| D16 |  |  |  |  | x |  |  |  | x | yes | yes | no | 11 | 5 | 3 | 1 |
| D19 |  |  |  |  | x |  |  |  | x | yes | yes | no | 12 | 3 | 3 | 1 |
| D20 |  |  |  |  | x |  |  |  | x | yes | no | yes | 6 | 2 | 3 | 1 |
| D22 |  |  |  |  | x |  |  |  | x | no | no | yes | 13 | 0 | 4 | 0 |
| D24 |  |  |  |  | x |  |  |  | x | yes | no | no | 11 | 7 | 3 | 3 |
| D25 |  |  |  |  | x |  |  |  | x | yes | yes | yes | 18 |  | 4 |  |
| D43 |  |  |  | P/D interface |  | x |  | x | x | yes | yes | yes | 15 | 3 | 4 | 1 |
| D45 |  |  |  | P/D interface | x | x |  | x | x | no | yes | yes | 11 | 2 | 3 | 1 |
| D46 |  |  |  | D | x | x |  | x | x | yes | yes | no | 14 | 6 | 4 | 2 |

**Supplementary Table 4. Overview of the patients included in the molecular phenotyping study.**

X indicates the analyses completed by each patient. P: palm, D: dorsum, AD: atopic dermatitis, W: week, mTLSS: modified Total Lesion Severity Score, IGA: Investigator Global Assessment. History of AD was defined as a diagnosis of AD including mild or intermittent forms, made prior to the diagnosis of CHE. Black squares indicate a premature end of study (no score available at week 16 and no paired sample).

**Supplementary Figure 1. Flow chart of the clinical study.** ITT = Intention-to-Treat

**Supplementary Figure 2. Evolution of Modified Total Lesion Symptom Score (mTLSS) subscores and Eczema Area and Severity Index (EASI) lower extremities score under dupilumab or placebo treatment from W0 to W16.**

Erythema, scaling, lichenification/hyperkeratosis, vesicles, edema, cracks and pruritus/pain, were assessed at weeks 0, 4, 8, 12 and 16 after treatment (A-G). The EASI lower extremities score (which partially reflects foot involvement) was assessed at weeks 0, 4, 8, 12, and 16 after treatment initiation (H). Data are presented as mean ± standard deviation. *p<0.05, **p<0.01, ***P<0.001. Wilcoxon test (A-H).

**Supplementary Figure 3.** Representative pictures of CHE evolution in four placebo-treated and four dupilumab-treated patients from baseline to week 16.

**Supplementary Figure 4. Baseline whole skin proteomic signature in CHE patients compared to healthy controls was restricted to a strongly compromised skin barrier.**

**A. Dendrogram of the hierarchical clustering of baseline CHE patients and healthy controls.** Patient numbers are reported at the bottom of the figure. CHE patients marked with a black rectangle below their number have a history of AD. **B. Number of DEG identified in each skin diseases related to CHE based on literature dataset.** Data of AD, classical psoriasis vulgaris (CP) and palmoplantar psoriasis (PPPso) patients are based on bulk-RNA sequencing whereas data of allergic contact dermatitis (ACD) and irritant contact dermatitis (ICD) patients are based on microarrays. All DEGs were identified compared to healthy controls. **C. DEGs overlap between AD, CP and CHE.** Venn diagram illustrating the percentage and number of overlapping DEGs between CHE (blue), AD (red) and CP (yellow). **D. PCA approach on all variable proteins obtained from LC-MS/MS shotgun proteomic in the skin, showing the segregation between CHE patients at baseline and healthy donor controls.** Patient and control numbers are reported above each sample. **E. Volcano plot representation of differentially expressed proteins (DEPs) between CHE patients and healthy controls at baseline.** Log2-FC threshold: < -1 and > 1, adjusted *P* value threshold < 0.05. **F. Hierarchically clustered heatmap representation of top 50 upregulated DEPs in CHE patients compared to controls.** Characteristic genes involved in skin barrier functions (green), antimicrobial defense (blue) or inflammation/ immune response processes (red) are highlighted. Red and blue represent up- and downregulated expression, respectively. CHE patients marked with a black rectangle below their number have a history of AD. **G. Top 30 most enriched pathways from Gene set enrichment analysis (GSEA) in CHE patients at baseline.** Y-axis represents ranked pathways, and X-axis shows the normalized enrichment score (NES). Blue to red colors represent low to high NES, respectively. The size of the bubbles is associated with the *P* value. Characteristic genes involved in skin barrier functions (green), antimicrobial defense (blue) or inflammation/ immune response processes (red) are highlighted. (A-D) n=4 controls and 6 baseline CHE patients.

**Supplementary Figure 5. Baseline CHE patients exhibited fewer alterations in circulating immune cells compared to AD or psoriasis controls.**

**A-B. Opt-sne concatenation of circulating lymphoid populations of baseline CHE patients compared to healthy controls.** (A) Relative expression of lymphoid biomarkers (CD19, CD56, CD57, TCRab, CD4, CD8 and CCR7) was assessed to determine lymphoid populations. Red and blue represent high and low expression levels of markers, respectively. (B) Distribution of major lymphoid populations in opt-sne. Biomarkers allow discriminating between B cells, NK cells, naive/TEMRA/TEM CD8^+^ T cells and naive/TEMRA/TEM CD4^+^ T cells. **C-D. Quantification of major circulating lymphoid populations in baseline CHE patients compared to healthy controls, AD and CP patients.** Percentage of each population among lymphoid cells (C) and conventional CD4^+^ T cells (D). DUMP = CD14, CD11c, CD1a, CD34. Data are presented as mean ± standard deviation. **P*<0.05, ***P*<0.01, ****P*<0.001, *****P*<0.0001. Mann–Whitney U test. n=14 controls, 19 baseline CHE patients, 5 AD patients and 6 CP patients. **E-F. Opt-sne concatenation of circulating dendritic cells populations of baseline CHE patients compared to healthy controls.** (E) Relative expression of lymphoid biomarkers (CD123, CD141, FCeRI, CD14, CD5) was assessed to determine dendritic cells populations. Red and blue represent high and low expression levels of markers, respectively. (F) Distribution of major lymphoid populations in opt-sne. Biomarkers allow discriminating between pDC, cDC1, CD5^-^ and CD5^+^ cDC2, CD14^-^ and CD14^+^ cDC3. **G. Quantification of major circulating dendritic cells populations in baseline CHE patients compared to healthy controls.** Percentage of each population among dendritic cells. DUMP = CD3, CD4, CD8, CD19. Data are presented as mean ± standard deviation. **P*<0.05. Mann–Whitney U test. n=14 controls, 28 baseline CHE patients. **H. Estimated abundancy of immune cells composition in the skin of baseline CHE patients compared to healthy controls using CIBERSORTx analysis.** **P*<0.05, ***P*<0.01, ****P*<0.001. n=25 healthy controls and 15 baseline CHE patients.

**Supplementary Figure 6. Gating strategy for lymphoid cells analysis by spectral flow cytometry.**

**Supplementary Figure 7. Gating strategy for myeloid cells analysis by spectral flow cytometry.**

**Supplementary Figure 8. At baseline, CHE patients exhibited a mixed immune activation profile in serum compared to healthy, AD, and psoriasis controls.**

**A. PCA approach on all variable proteins obtained from LC-MS/MS shotgun proteomic in serum, showing the segregation between CHE patients at baseline and healthy donor controls.** Patient and control numbers are reported above each sample. **B. Volcano plot representation of differentially expressed proteins (DEPs) between CHE patients and healthy controls.** Log2-FC threshold: < -1 and > 1, adjusted *P* value threshold < 0.05. **C. Top 30 most enriched pathways from Gene set enrichment analysis (GSEA) in CHE patients.** Y-axis represents ranked pathways, and X-axis shows the normalized enrichment score (NES). Blue to red colors represent low to high NES, respectively. The size of the bubbles is associated with the *P* value. Characteristic genes involved in skin barrier functions (green), antimicrobial defense (blue) or inflammation/ immune response processes (red) are highlighted. (E-G) n=4 controls and 7 baseline CHE patients. **D. Expression of selected AD, CP, and ACD biomarkers in serum comparing CHE patients at baseline, healthy controls, AD, and CP patients.** Protein levels were quantified using NULISA Protein Quantification (NPQ). CHE baseline patients are shown in black, healthy controls in blue, AD patients in red, and CP patients in yellow. **P*<0.05, ***P*<0.01. Wilcoxon test. CHE baseline (n=35), healthy controls (n=6), AD (n=7), CP (n=7).

**Supplementary Figure 9. DEGs comparison between dupilumab-treated CHE and AD patients in the skin.**

**A. PCA approach on all variable genes obtained from bulk-RNA sequencing, showing no segregation between baseline CHE patients and placebo-treated CHE patients.** Patient numbers are reported above each sample. **B.** **Dendrogram of the hierarchical clustering of baseline CHE patients and placebo-treated CHE patients.** Patient numbers are reported at the bottom of the figure. CHE patients marked with a black rectangle below their number have a history of AD. **C. Volcano plot representation of DEGs identified in placebo-treated CHE patients compared to baseline.** Log2-FC threshold: < -1 and > 1, *P* value threshold < 0.05. (F-H) n=15 baseline CHE patients and 5 placebo-treated CHE patients. **D. Representation of the top DEGs from CHE and AD patients treated with dupilumab for 16 and 12 weeks respectively, each compared to their respective baselines.** A Venn diagram identified 44 common DEGs, 447 CHE-specific DEGs, and 149 AD-specific DEGs. The top 20 DEGs with the highest absolute log2 fold change were selected among the common genes and for each disease-specific gene set. The size of the bubbles is related to log2 fold change while the color reflects downregulation or upregulation, in green and red respectively. **E. Representation of genes belonging to the keratinocyte differentiation GSEA pathway in baseline AD, CP and CHE and evolution under treatment with dupilumab.** The size of the bubbles is related to log2 fold change while the color reflects downregulation or upregulation, in green and red respectively.

**Supplementary Figure 10. Skin barrier defect proteomic signature was restored after 16 weeks-dupilumab treatment compared to placebo** **in CHE patients.**

**A. PCA approach on all variable proteins obtained from LC-MS/MS shotgun proteomic in the skin, showing the segregation between baseline CHE patients and dupilumab-treated CHE patients.** Patient numbers are reported above each sample. **B. Volcano plot representation of DEPs in dupilumab-treated CHE patients compared to baseline.** Log2-FC threshold: < -1 and > 1, adjusted *P* value threshold < 0.05. **C.** **Hierarchically clustered heatmap of the top 50 up- and downregulated DEPs in dupilumab-treated CHE patients compared to baseline.** Characteristic genes involved in skin barrier functions (green), antimicrobial defense (blue) or inflammation/ immune response processes (red) are highlighted. Patient numbers are reported at the bottom of the figure. CHE patients marked with a black rectangle below their number have a history of AD. Red and blue represent up- and downregulated expression, respectively. **F. Top 30 most significantly enriched pathways from GSEA in dupilumab-treated CHE patients compared to baseline.** Y-axis represents ranked pathways, and X-axis shows NES. Blue to red colors represent low to high NES, respectively. The size of the bubbles is related to the *P* value. Characteristic genes involved in skin barrier functions (green), antimicrobial defense (blue) or inflammation/ immune response processes (red) are highlighted. (A-D) n=6 baseline CHE patients and 3 dupilumab-treated CHE patients. **E. PCA approach on all variable proteins obtained from LC-MS/MS shotgun proteomic in the skin, showing no segregation between baseline CHE patients and placebo-treated CHE patients.** Patient numbers are reported above each sample. N=6 baseline CHE patients and 2 placebo-treated CHE patients.

**Supplementary Figure 11. No major change was observed in circulating immune cells in dupilumab-treated patients compared to placebo at week 16 in CHE patients.**

**A-C. Distribution of major circulating lymphoid populations in CHE patients treated with dupilumab or placebo.** Opt-sne concatenation representation (A) and percentage of each population among lymphoid cells or conventional CD4^+^ T cells at week 0 and 16 in (B) placebo-treated and (C) dupilumab-treated CHE patients. DUMP = CD14, CD11c, CD1a, CD34. N=9 at week 0 (W0) and 7 at week 16 (W16) for placebo, and 10 at week 0 and 7 at week 16 for dupilumab-treated CHE patients. Data are presented as mean ± standard deviation. (B,C) ns: not significant. Mann–Whitney U test. **D-F. Distribution of major circulating dendritic cells populations in CHE patients treated with dupilumab or placebo.** Opt-SNE concatenation representation (A) and percentage of each dendritic cells subset at week 0 and 16 in (E) placebo-treated and (F) dupilumab-treated CHE patients. DUMP = CD3, CD4, CD8, CD19. n=13 at week 0 (W0) and 9 at week 16 (W16) for placebo, and 13 at week 0 and 11 at week 16 for dupilumab-treated CHE patients. Data are presented as mean ± standard deviation. (E, F) Mann–Whitney U test. **G. Changes in the estimated abundance of immune cell populations in the skin of dupilumab-treated CHE patients, compared to placebo-treated and baseline CHE patients, as assessed by CIBERSORTx analysis.** **P*<0.05, ***P*<0.01, ****P*<0.001. n=15 baseline CHE patients, n=8 dupilumab-treated CHE patients and n=5 placebo-treated CHE patients.

**Supplementary Figure 12. Decreased type 2 and type 3 immune responses observed in dupilumab-treated CHE patients in serum.**

**A. PCA approach on all variable proteins obtained from LC-MS/MS shotgun proteomic in serum, showing no segregation between baseline CHE patients and dupilumab-treated CHE patients.** Patient numbers are reported above each sample. **B. Volcanoplot representation of DEPs in dupilumab-treated CHE patients compared to baseline.** Log2-FC threshold: < -1 and > 1, adjusted *P* value threshold < 0.05. **C. PCA approach on all variable proteins obtained from LC-MS/MS shotgun proteomic in serum, showing no segregation between baseline CHE patients and placebo-treated CHE patients.** Patient numbers are reported above each sample. N=6 baseline CHE patients and 2 placebo-treated CHE patients. **D. Volcano plot representation of DEPs in placebo-treated CHE patients compared to baseline (untargeted shotgun proteomics).** Log2-FC threshold: < -1 and > 1, adjusted *P* value threshold < 0.05. **E. Volcano plot representation of DEPs in CHE patients treated with placebo for 16 weeks compared to their baseline (NULISAseq).** Protein expression was quantified using the NULISAseq™ Inflammation Panel 250. DEPs were defined by a log2 fold-change threshold of > 0.5 or < -0.5 and an adjusted *P* value < 0.05. **F. Expression of selected AD, CP, and ACD biomarkers in serum of CHE patients treated with dupilumab or placebo compared to baseline.** CHE patients at baseline are shown in black, dupilumab-treated patients in red, placebo-treated patients in gray. Protein levels were quantified using NULISA Protein Quantification (NPQ). **P*<0.05. Wilcoxon test. CHE baseline (n=35), dupilumab-treated CHE (n=20), placebo group (n=15).
